# DCE@urLAB: a dynamic contrast-enhanced MRI pharmacokinetic analysis tool for preclinical data

**DOI:** 10.1186/1471-2105-14-316

**Published:** 2013-11-04

**Authors:** Juan E Ortuño, María J Ledesma-Carbayo, Rui V Simões, Ana P Candiota, Carles Arús, Andrés Santos

**Affiliations:** 1 CIBER de Bioingeniería, Biomateriales y Nanomedicina (CIBER-BBN), 50018 Zaragoza, Spain; 2 Biomedical Image Technologies Group, Departamento de Ingeniería Electrónica, Universidad Politécnica de Madrid, 28040 Madrid, Spain; 3 Department of Maternal-Fetal Medicine (ICGON), Fetal and Perinatal Medicine Research Group (IDIBAPS), Hospital Clínic, Universitat de Barcelona, Sabino de Arana 1- Helios III, 08028 Barcelona, Spain; 4 Departament de Bioquímica i Biologia Molecular, Unitat de Biociències, Universitat Autònoma de Barcelona, Edifici Cs, Campus UAB, 08193 Cerdanyola del Vallès, Spain; 5 Institut de Biotecnologia i de Biomedicina Vicent Villar Palasí (IBB), Universitat Autònoma de Barcelona, Edifici Cs, Campus UAB, 08193 Cerdanyola del Vallès, Spain

**Keywords:** DCE-MRI, Imaging, Levenberg-Marquardt, Fitting, Preclinical, Pharmacokinetics, Animal models, High field MR, IDL

## Abstract

**Background:**

DCE@urLAB is a software application for analysis of dynamic contrast-enhanced magnetic resonance imaging data (DCE-MRI). The tool incorporates a friendly graphical user interface (GUI) to interactively select and analyze a region of interest (ROI) within the image set, taking into account the tissue concentration of the contrast agent (CA) and its effect on pixel intensity.

**Results:**

Pixel-wise model-based quantitative parameters are estimated by fitting DCE-MRI data to several pharmacokinetic models using the Levenberg-Marquardt algorithm (LMA). DCE@urLAB also includes the semi-quantitative parametric and heuristic analysis approaches commonly used in practice. This software application has been programmed in the Interactive Data Language (IDL) and tested both with publicly available simulated data and preclinical studies from tumor-bearing mouse brains.

**Conclusions:**

A user-friendly solution for applying pharmacokinetic and non-quantitative analysis DCE-MRI in preclinical studies has been implemented and tested. The proposed tool has been specially designed for easy selection of multi-pixel ROIs. A public release of DCE@urLAB, together with the open source code and sample datasets, is available at http://www.die.upm.es/im/archives/DCEurLAB/.

## Background

Dynamic contrast-enhanced magnetic resonance imaging (DCE-MRI) involves the acquisition of sequential images in rapid succession during and after the intravenous administration of a, usually, low-molecular weight contrast agent (CA), which includes a paramagnetic component such as gadolinium (Gd^3+^). This functional imaging modality has proven to be useful in tumor differentiation, being a sensitive marker of antiangiogenic treatment effect [1,2].

When T_1_-weighted magnetic resonance (MR) sequences are used, the CA induces a signal enhancement related with the shortening of spin-lattice or longitudinal relaxation time (T_1_), the time course of which can be related to physiological parameters. The most common CA used in T_1_-weighted DCE-MRI, Gadolinium-diethylenetriamine penta-acetic acid (Gd-DTPA), is able to transverse the vascular endothelium (except when the blood-brain barrier is intact) and enter the extravascular-extracellular space (EES), but is unable to cross the cellular membrane. Thus, in DCE-MRI the measured signal intensity changes derive mostly from CA that extravasates to the EES [3,4]. The dynamics of exchange between the capillary bed and the EES can be evaluated and are usually modeled as an open two-compartment model, dependent on the washout rate between EES and plasma (*k*_*ep*_), and the volume transfer constant between plasma and EES, denoted as *K*^*trans*^[5].

DCE-MRI has been used to investigate permeability and perfusion in small animal tumor models [6,7]. A key consideration in rodents is that the concentration of CA in vascular plasma evolves rapidly compared to tissue, and is quite difficult to sample the maximum signal intensity to effectively characterize the tissue pharmacokinetics. Since sampling the blood (the gold standard in humans) is very invasive in small animals, kinetic models that do not rely on arterial input function (AIF) measurements are desirable in preclinical DCE-MRI.

Therefore, the software application presented in this manuscript is aimed at filing this gap and providing a powerful and versatile T_1_-weighted DCE-MRI processing tool, and at the same time, intuitive and easy-to-use in preclinical studies. It has been implemented in Interactive Data Language (IDL), accessible at http://www.exelisvis.com/idl.

The DCE@urLAB application integrates pixel-wise pharmacokinetic analysis using the following models: Tofts [8], Hoffmann [9], Larsson [10], and a reference region (RR) model [11]. The Tofts pharmacokinetic model has been widely applied to characterize murine tumors [12-14], as well as the Hoffmann pharmacokinetic model [15,16]. The Larsson model has not been extensively applied to small animal DCE-MRI, but is the third model typically used in theoretical studies and reviews [5,17]. Finally, the RR model has been proposed as an alternative when AIF cannot be precisely estimated.

### Existing software

Model-based and semi-quantitative analysis of T_1_-weighted DCE-MRI can be performed with general purpose pharmacokinetic compartmental analysis packages, either non-commercial, like WinSAAM [18], JPKD [19], or commercial, like SAAM II [20]. These are complex tools that require specific training and need to be adjusted to the particular problem of DCE-MRI. Pixel-wise analysis and ROI selection of images are also not included in these platforms.

Among the software specifically designed for DCE-MRI data are the packages BioMap [21], PermGUI and PCT [22], Toppcat [23], DcemriS4 [24], and DATforDCEMRI [25].

BioMap is built in IDL, and supports compartmental analysis over ROIs through the perfusion tool. Two ROIs must be defined, one describing the CA tissue-concentration and the other the concentration of the CA in blood plasma (*C*_*p*_). When *C*_*p*_ cannot be measured in an ROI, either because the image does not contain a large blood vessel, or the signal from the blood vessel is corrupted by pulsation, movement or saturation effects, a theoretical bi-exponential decay function can be used as *C*_*p*_. Published results with DCE-MRI using BioMap include small animal studies [12,26,27]. Although BioMap can generate pixel maps, it does not work with coarse resolutions and is limited to the Tofts model, with a bi-exponential model of *C*_*p*_.

PermGUI and PCT [22] are freeware applications oriented to extract the permeability coefficient of the blood brain barrier (BBB) in human patients. The tools analyze DCE-MRI images using the Patlak model [28]. This model is also used in the package Toppcat, which runs as a plugin of ImageJ [29]. Toppcat is also free of charge for educational and research purposes.

DcemriS4 [24] is a collection of shell scripts to help automate the quantitative analysis of DCE-MRI and diffusion weighted imaging (DWI), and written in the R programming environment [30]. Kinetic parametric estimation is performed with the Tofts model and non-linear regression, Bayesian estimation or deconvolution algorithms. AIF is parameterized with a tri-exponential function [31] to obtain an analytical solution of the convolution integral and increase computational efficiency.

DATforDCEMRI [25] is an R package tool which allows performing kinetic deconvolution analysis [32] and visualizing the resulting pixel-wise parametric maps. Like DcemriS4, this software package requires an end-user training in R programming environment.

These software packages are primarily designed for human studies and thus are not well suited for some typical requirements of preclinical DCE-MRI, e.g., the difficulty in accurately measuring the AIF in small animals makes that typical models in human studies cannot be used and ultimately requires the use of the Hoffmann or RR models. These models are not implemented in available software packages. Other important functionalities such as the difficulty in reading the imaging format produced by preclinical studies prevent from the use of those packages by the preclinical research community. Thus, in-house solutions are commonly used in DCE-MRI small animal studies, using Matlab programming environment [33,34], LabView [35] or IDL [36-39], but they are mostly designed for a specific study and with limited availability.

## Implementation

In this section, the compartmental models implemented in the DCE@urLAB analysis tool are described. Additional information and technical details can be found in the “DCEurLAB Methods.pdf” document included in the software package, accessible at http://www.die.upm.es/im/archives/DCEurLAB/ and in the Additional file 1. This section also includes a brief description of the graphical user interface (GUI) usage.

### DCE-MRI pharmacokinetic modeling

**Figure 1:**
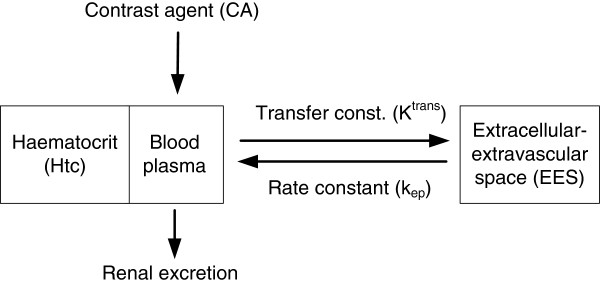
**Pharmacokinetic model.** Body compartments accessed by low molecular weight CA injected intravenously (e.g., Gd-DTPA).

Model-based pharmacokinetic analysis of T_1_-weighted DCE-MRI used in the DCE@urLAB application tool is open bi-compartmental, representing the blood plasma and the EES, and assume some basic concepts in tracer kinetics and MR [5]. As the CA does not enter the intracellular space, this compartment is not considered in the model. The blood plasma is associated with the central compartment, the wash-out to the kidneys and the intake from the injected contrast, while the EES is the peripheral compartment. This compartmental scheme is shown in Figure 1. We should note that a bi-compartmental model does not consider the complex biology of the tumor. Although multi-compartment models have been proposed [40], the open bi-compartmental model has been able to fit DCE-DRI data surprisingly well and is therefore widely accepted by the research community. Time-course changes in tissue CA concentration are modeled as a result of first-order exchange of the CA molecules between compartments. A modified general rate equation [41] describes the CA accumulation and wash-out rate in the EES, under the assumption that the CA is well-mixed in the blood plasma:

(1) $$\frac{dC_{e}(t)}{\text{dt}}=\frac{K^{\text{trans}}}{v_{e}}\left(C_{p}(t)-C_{e}(t)\right),\;\;\;v_{p}C_{p}+v_{e}C_{e}=C_{t}$$

where *C*_*e*_ is the CA concentration in EES, *C*_*t*_ is the total CA concentration in the tissue, *v*_*p*_ is the fractional volume of blood plasma, and *v*_*e*_=*K*^*trans*^/*k*_*ep*_ is the fractional volume of EES. The physiological meaning of *K*^*trans*^ depends on the biological mechanism of CA exchange (i.e., blood flow, permeability, or a mixed case). If no prior information about the tissue is available, then is prudent to leave the interpretation open.

#### Tofts model

The Tofts pharmacokinetic model [8,42] is derived from Equation 1, excluding the contribution from vascular plasma. Tofts originally proposed a bi-exponential model for *C*_*p*_. In that case, the solution of Equation 1 for an instantaneous bolus injection reduces to:

(2) $$\begin{array}{ll} C_{t}(t) & =DK^{\text{trans}}\overset{2}{\underset{i=0}{\sum }}a_{i}\frac{e^{-K^{\text{trans}}t/v_{e}}-e^{-m_{i}t}}{m_{i}-K^{\text{trans}}/v_{e}},\\ C_{p}(t) & =D\overset{2}{\underset{i=0}{\sum }}a_{i}e^{-m_{i}t} \end{array}$$

from which *K*^*trans*^ and *v*_*e*_ can be estimated through a minimization algorithm. Amplitudes *a*_*i*_ and time constant *m*_*i*_ are estimated from a population average and *D* is the injected CA dose. The extended or modified Tofts model [5] corresponds to the adding of the contribution of the blood plasma fraction *v*_*p*_*C*_*p*_(*t*) to account for the tracer in the vasculature. In this case, the unknown parameters are *K*^*trans*^, *v*_*e*_, and *v*_*p*_. The discrete approximation measured, or population averaged vascular plasma CA concentrations at sampling times, can be solved with least-squares minimization methods, e.g., using the matrix-vector formulation of the discrete convolution:

(3) $$C_{t}(t)=C_{p}(t)*\left(K^{\text{trans}}e^{-k_{\text{ep}}t}\right)$$

The Tofts model produces reliable results if the tissue is weakly vascularized, while the extended Tofts model can also be applied to highly perfused tumors [43]. It is important to note that the quantification of Tofts parameters requires the estimation *C*_*p*_(*t*) from the acquired MR signal. Thus, an additional MRI model has been included in the DCE@urLAB application and is discussed later.

#### Hoffmann model

The Hoffmann model [9] is derived from the Brix model [44] for fast bolus injection, and assumes that the CA transfer from blood plasma to EES is a slow process. The model establishes a direct relationship between MR signal enhancement and CA exchange rates, without the need for AIF estimation and MR quantification. After the bolus injection, the model is described as:

(4) $$\frac{S(t)}{S_{0}}=1+A^{H}k_{\text{ep}}\left(\frac{e^{-k_{\text{ep}}t}-e^{-k_{\text{el}}t}}{k_{\text{el}}-k_{\text{ep}}}\right)$$

where *S*(*t*) is the MR signal course from tissue and *S*_0_ is the MR signal before CA injection. The fitting parameters are: *k*_*ep*_; *A*^*H*^, which approximately corresponds to the size of the EES; and *k*_*el*_, the renal elimination constant.

#### Larsson model

The Larsson model [10] uses a known blood plasma CA concentration course, either measured from blood samples or estimated from the MRI data. It is assumed that the MR signal is linearly related to the CA concentration. In that case, the MR signal is modeled as:

(5) $$S(t)=S_{0}+\left(\frac{\dot{S}(t)}{\overset{N}{\underset{i=0}{\sum }}a_{i}}\right)\overset{N}{\underset{i=0}{\sum }}a_{i}\frac{e^{-k_{\text{ep}}t}-e^{-m_{i}t}}{m_{i}-k_{\text{ep}}}$$

where $\dot{S}(t)$ is the initial slope of the MR signal and *S*_0_ the MR signal value prior to CA injection. *C*_*p*_ is approximated as a sum of N exponentials with amplitudes *a*_*i*_ and time constant *m*_*i*_.

#### RR model

An alternative to a populations-based or estimated AIF, is the RR model [11]. The approach uses a well-characterized tissue to combine two versions of Equation 1, one for the RR and another one for the tissue of interest. This allows the removal of *C*_*p*_ in the solution of the resulting equation [11].

(6) $$\begin{array}{ll} C_{t}(t) & =\frac{K^{\text{trans}}}{K^{\text{trans},r}}\left(C_{t,r}(t)+\left(\frac{K^{\text{trans},r}}{v_{e,r}}-\frac{K^{\text{trans}}}{v_{e}}\right)\right)\\ & \;\times \left(\int_{0}^{t}C_{t,r}\left(t^{\prime }\right)e^{-K^{\text{trans}}\left(t-t^{\prime }\right)/v_{e}}dt^{\prime }\right) \end{array}$$

where *C*_*t*,*r*_ is the concentration of CA in the RR tissue and *K*^*trans*,*r*^ and *v*_*e*,*r*_ are the quantitative parameters for the RR.

#### MRI model

The Tofts and RR models require the calibration of CA concentration from measured MRI parameters. If the bulk magnetic susceptibility (BMS) shift is negligible, the relationship between T_1_ and CA concentration is determined by the Solomon-Bloembergen equation [45]:

(7) $$\frac{1}{T_{1}(t)}=\frac{1}{T_{10}}+r_{1}C_{t}(t)$$

where *T*_10_ is the T_1_ value before CA injection and *r*_1_ is the longitudinal relaxivity. The relationship between CA concentration and the relative increase in signal intensity can be derived from the Bloch equations for any imaging sequence, e.g., the signal for a T_1_-weighted spin-echo pulse sequence (at short echo time) with repetition time (TR) is:

(8) $$S(t)=S_{0}\left(1-e^{\text{TR}/T_{1}(t)}\right)$$

From Equation 7 and 8, *C*_*t*_ is equal to:

(9) $$\;C_{t}(t)=\frac{1}{r_{1}}\left(\frac{1}{\text{TR}}\ln\left(\frac{S_{0}}{S_{0}-S(t)\left(1-e^{-\text{TR}/T_{10}}\right)}\right)-\frac{1}{T_{10}}\right)$$

For spoiled gradient-echo pulse sequences with flip angle *α*, the MR signal is equal to:

(10) $$S(t)=\left(\frac{S_{0}\left(1-e^{-\text{TR}/T_{1}(t)}\right)\mathrm{sin\alpha}}{1-e^{-\text{TR}/T_{1}(t)}\mathrm{cos\alpha}}\right)$$

The signal intensity is converted to CA concentration in tissue using the equation from [46] to calculate *T*_1_(*t*):

(11) $$\;\begin{array}{rl} {T_{1}(t)}^{-1} & =-\frac{1}{\text{TR}}\ln\left(\frac{1-\left(\frac{S(t)-S_{0}}{S_{0}\mathrm{sin\alpha}}+\frac{1-m}{1-m\cos\alpha }\right)}{1-\left(\frac{S(t)-S_{0}}{S_{0}\mathrm{sin\alpha}}+\frac{1-m}{1-m\cos\alpha }\right)\mathrm{cos\alpha}}\right),\\ m & =e^{-\text{TR}/T_{10}} \end{array}$$

and CA concentration in tissue is calculated from Equation 7. Note that *r*_1_ and T_10_ must be known to quantify the tissue concentration from the MR signal. T_10_ may be estimated using the ratio of two spin-echo images collected with different TR. The estimation error can be reduced with a higher number of images with a least-squares minimization algorithm.

### Estimation of model parameters

Curve fitting routines have been implemented using internal IDL functions and the freely available MPFIT IDL library [47]. MPFIT contains a set of non-linear regression algorithms for robust least-squares minimization, based on the freely available MINPACK package (Univ. of Chicago, http://www.netlib.org/minpack/) a library of FORTRAN subroutines for solving nonlinear equation systems.

DCE@urLAB uses the Levenberg-Marquardt algorithm (LMA) [48] to perform the non-linear least squares regression in each pixel of the analyzed ROI. LMA has demonstrated robustness in the pharmacokinetic modeling of DCE-MRI [49]. LMA is used to estimate appropriate parameters in several models: Tofts (with bi-exponential *C*_*p*_ of Equation 2 or solving the discrete convolution Equation 3); the equivalent extended Tofts model; Hoffmann (Equation 4); Larsson (Equation 5); and also the RR model (Equation 6).

Pixel-based processing of dynamic MRI data can be demanding in terms of memory and CPU, and hardware requirements will vary depending on the size of data sets, as well as the number of pixels selected. In any case, it is recommended to run the program in systems with at least 2 GB of RAM memory. In addition to pharmacokinetic modeling, model-free semi-quantitative analysis can be performed, including IAUC (initial area under curve), RCE (relative contrast enhancement) and TTM (time to max enhancement) [50].

### Description and use of the GUI

**Figure 2:**
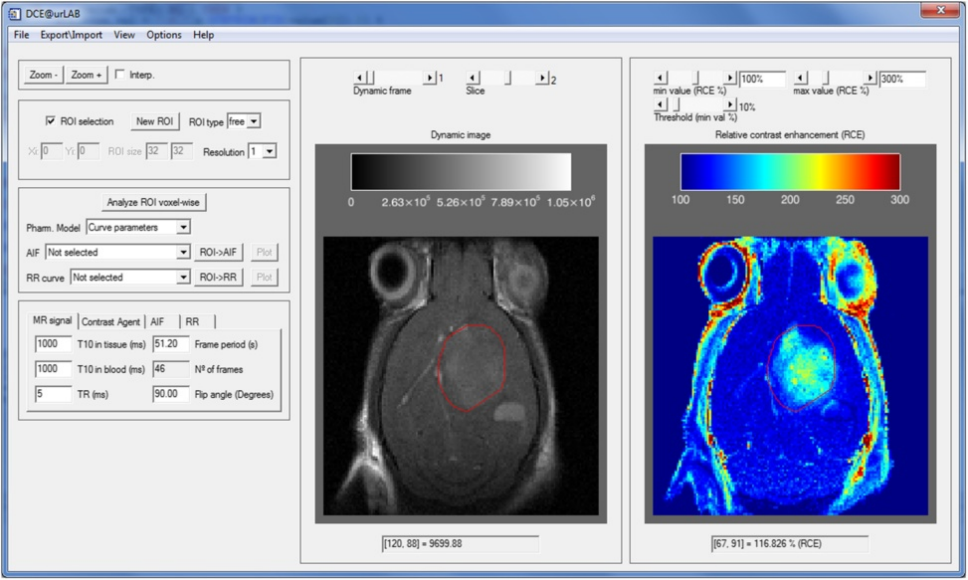
**Main window interface.** In this example, a mouse brain tumor study is displayed: GL261 glioblastoma (see also the Results section). The main window interface is shown before the ROI has been selected. In the right side of the window, the RCE image is drawn with a “rainbow” color palette. The DCE-MRI image is drawn with “black & white” color palette in the central part. The Upper slides allow changing the current time frame and Z-slice. Other tabs, such as zoom options, ROI selection options, parametric selection and initial constants, are grouped in the left side of the main window interface.

The DCE@urLAB GUI is composed of a main window, which opens when the tool is executed, and auxiliary windows for results, input/output processes, or auxiliary activities. Figure 2 shows an appearance of the main window once the DCE-MRI study is loaded in memory. The complete and detailed functionality of the GUI is described in the user manual included in the downloadable software package. A general overview is presented in this section.

#### Input data

The software tool accepts DCE-MRI sequences and auxiliary inputs: T_10_ maps, AIF data and pre-calculated ROIs. Interface functionality is disabled until a 4-dimensional DCE-MRI study is open. The tool considers the sequence set to be a 4D stack of images in X-Y-Z-time order. Data can be imported from DICOM format, Bruker Biospin MRI data format (http://www.bruker.com/products/mr/mri.html), as well as from binary unformatted data. If the dynamic MR sequence is loaded properly, the interface will show a single 2D slice of the whole 4D data set in the left display tab, and a relative contrast enhancement (RCE) image in the right display tab (Figure 2).

The platform is specially designed to perform ROI or pixel-wise analysis over the selected ROI belonging to a single slice in the Z dimension (Z-slice for short). These ROIs can be exported in a custom format and subsequently imported in another work session. When required for a specific MRI model, T_10_ maps can be loaded from the menu file tab. AIF data can also be imported from previously saved sessions or external acquisitions.

#### Displaying data sets

After loading a valid DCE-MRI sequence, main processing options and menus will become activated. The user is now able to select ROIs, change parameters, as well as configure visualization options. Nevertheless, other options will not be activated until a valid ROI is drawn or imported.

The user can navigate through dynamic frames or Z-slices to select an active ROI for the pharmacokinetic analysis. The color palette of both MRI and RCE displays can be changed by selecting this option on the menu bar (options drop down menu). The user can additionally change the brightness, contrast, alpha channel, etc. Pressing mouse buttons on the display images produces different actions depending on the ROI selection mode. When the ROI selection mode is not activated, the actions allowed are: 

•Pressing the right mouse button on any image will plot the dynamic MR signal course of the pointed pixel.

•If the left mouse button is pressed over the MRI window, the value of the current pixel appears in the information label located at the bottom of the MRI window tab.

•When the left mouse button is pressed on the RCE image, the RCE value (%) of the current pixel will be shown in the associated information label.

#### Selecting and defining ROIs

If the ROI selection mode is activated, right and left mouse buttons are used to manually place ROIs in the selected slice. The ROI types can be Box, Full or Free-drawing type. The ROI definition depends on the type of ROI selected. If a Box-type is selected, the upper left and bottom right corners of the ROI are defined by pressing the left mouse button over the image, or alternatively, typing their X and Y coordinates in editable text fields. If the Full ROI type is selected, the current Z-slice is then defined as a ROI. In the Free-drawing ROI type, the user moves the pointer while pressing down the left mouse button over the image to manually delineate the contour of the ROI. The ROI can be deleted in every moment using the New ROI button and starting again. Finally, the user must also choose the resolution in the Z-slice, i.e., select the pixel size for processing options. The finest resolution corresponds to the intrinsic resolution of the image, but the user can also select coarser resolutions from 2 ×2 to 10 ×10 pixels in the Z-slice (x-y plane). This option allows a direct comparison with other applications using low-resolution maps. The selected ROIs are currently limited to a single Z-slice.

#### Input parameters

Processing input parameters should be checked before each ROI analysis to obtain accurate results. Input parameters are organized in tabs (located on the lower-right of the main window interface). Each tab groups a set of related parameters. The MR signal tab contains MRI data related constants (e.g., frame period, repetition time, etc.). The AIF tab groups the parameters used in the bi-exponential model for the CA concentration in blood plasma proposed by Tofts. The CA tab must be completed with information concerning the injected contrast (e.g., injection frame, relaxivity, injected dose, etc.). Finally, the RR tab contains additional data used in the reference region model. These input parameters will be used or not depending on the pharmacokinetic study selected, e.g., the AIF tab is only read when the Tofts model is applied.

#### Pharmacokinetic processing and analysis

**Figure 3:**
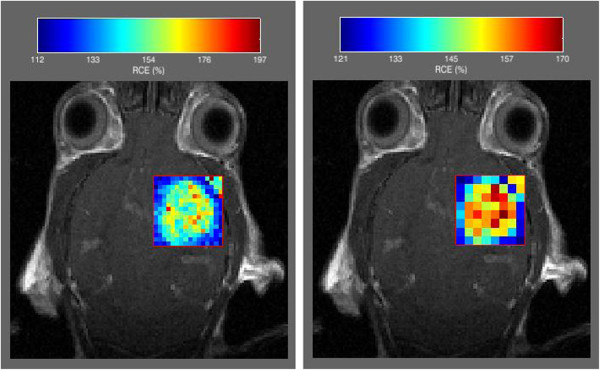
**Pixel resolution.** The pixel where pharmacokinetic modeling is performed can vary in resolution: From intrinsic image resolution (the finest) to coarse resolution. In the figure, two different coarse resolutions are shown for a mouse GL261 glioblastoma.

Pharmacokinetic models are estimated by pressing the Analyze ROI button. Note that this option is inactive until a valid ROI has been previously drawn or imported. Hoffmann, Tofts (standard and extended), Larsson and RR models can be selected for analysis. Model-free parameters (i.e., semi-quantitative parameters) are included as an independent option. Analytical or numerical solutions of the convolution integral are automatically chosen depending on the type of AIF loaded. Once the analysis is finished, the user can select the parameter to be displayed or saved in disk, by using the drop lists associated to each pharmacokinetic model. An example of the result with Box-type ROIs and two different resolutions is shown in Figure 3. The visualization menu located on the left (Figure 2) can select the transparency and scale of the parametric map.

**Figure 4:**
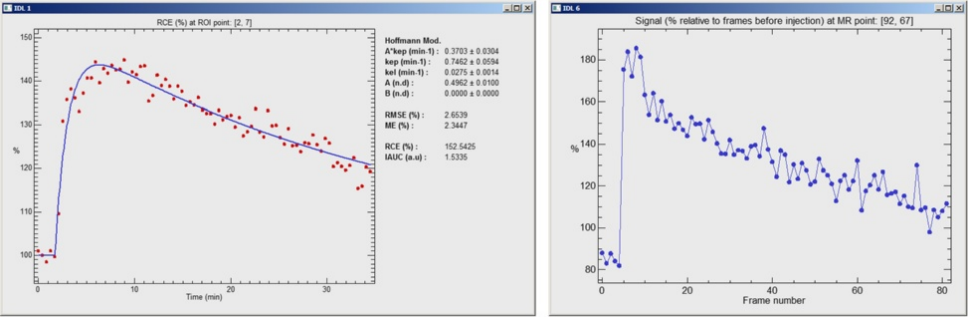
**Modeled and acquired DCE-MRI curves.** Modeled (left) and acquired (right) DCE-MRI curves. The software tool can plot the time-course changes in individual pixels or in the whole ROI. The curve can be compared with the analytic pharmacokinetic model (left plot), where the acquired data are represented as dots and the fitted evolution as a continuous curve.

The software tool also provides detailed information of the estimated pharmacokinetic model at pixel level; if the left mouse button is pressed when the pointer is located over the ROI, the adjusted curve of the parametric model associated to the selected pixel is plotted together with the DCE-MRI sequence values. An example of this plot is shown in Figure 4. The plot represents the model curve with the estimated parameters displayed on the right side.

Complementary results and data can be accessed from the menu bar, e.g., in the Export/import drop-down menu, several options can be selected to export images shown on the screen, ROI kinetics, or the set of parametric values of the selected ROI. Single column, multiple column, and matrix format are available.

## Results

### Validation using simulated data

**Figure 5:**
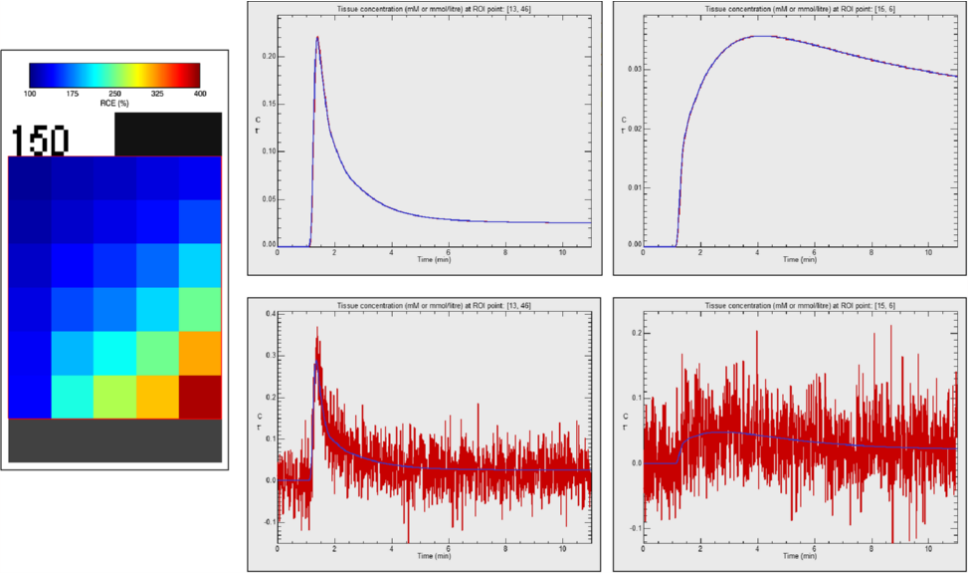
**QIBA test data corresponding to the Tofts model.** Left: RCE values of 30 combinations of *K*^*trans*^ and *v*_*e*_ values of simulated QIBA test data without added noise. Upper-right: curve-fitting with the Tofts model over two random points of the QIBA test data without noise. Lower-right: curve-fitting of the Tofts model adding Gaussian noise of zero mean and *σ*=20% of the signal baseline.

Tofts and extended Tofts models have been validated with the Quantitative Imaging Biomarkers Alliance (QIBA) DCE-MRI synthetic data, which are publicly available at http://dblab.duhs.duke.edu. The physiologic model is described in [51] and was simulated using JSIM [52]. Two sets of DCE-MRI images were used, corresponding to the Tofts model and the extended Tofts model. Data is available in DICOM part 10 format. Simulation parameters of the Tofts model were: Flip angle, 30°; TR, 5 ms; time interval between frames, 0.5 s; T_10_ in tissue, 1000 ms; T_10_ in blood vessels, 1440 ms; Haematocrit, 45%. A 10 minute study was simulated, with injection of CA occurring at 60 s. The data in the test images was generated using several combinations of *K*^*trans*^ and *v*_*e*_. *K*^*trans*^ takes values {0.01, 0.02, 0.05, 0.1, 0.2, 0.35} *min*^−1^ and *v*_*e*_ takes {0.01, 0.05, 0.1, 0.2, 0.5}. The image frames contain 10 ×10 pixels patches of each and combination. The vascular region was located in the bottom strip of the image. An RCE image is shown in Figure 5.

**Figure 6:**
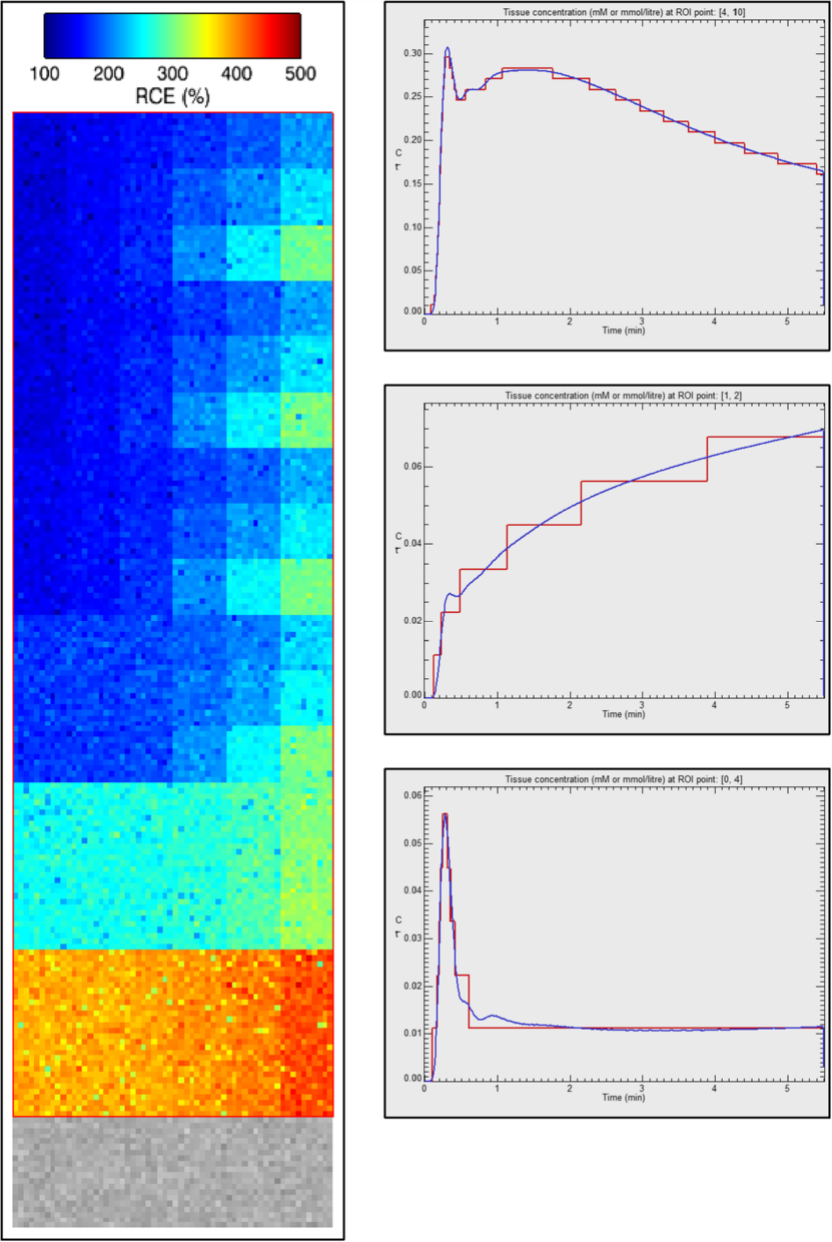
**QIBA test data corresponding to the extended Tofts model.** Left: RCE values of 108 parameter combinations, adding Gaussian noise of zero mean and *σ*=20% of signal prior to CA injection. Right: Extended Tofts model fitting over three random ROIs where each ROI comprises a single 10 ×10 pixels box with common parameters.

The extended Tofts model data have the following parameters: Flip angle, 25°; TR, 5 ms; time interval between frames, 0.5 s; T_10_ in tissue, 1000 ms; T_10_ in blood vessels, 1440 ms; Haematocrit, 45%. A 3.5 min study is simulated, with injection of CA occurring at 5 s. The data were generated using combinations of *K*^*trans*^, *v*_*e*_ and *v*_*p*_; *K*^*trans*^ varies over {0, 0.01, 0.02, 0.05, 0.1, 0.2} *min*^−1^, *v*_*e*_ takes values {0.1, 0.2, 0.5}, while *v*_*p*_ takes {0.001, 0.005, 0.01, 0.02, 0.05, 0.1}. Each combination of these three parameters is contained in a 10 ×10 pixel patch. The vascular region is the bottom 60 ×20 pixels strip of the image. An RCE image of this test data is represented in Figure 6. The kinetic variation of three different combinations of parameters is also shown in Figure 6. It can be appreciated that the discretization uncertainty in this data set is larger than in the former data set (Figure 5), and it is due to the lower value of equilibrium magnetization used in the simulation.

#### Results with Tofts model applied to QIBA test data

**Figure 7:**
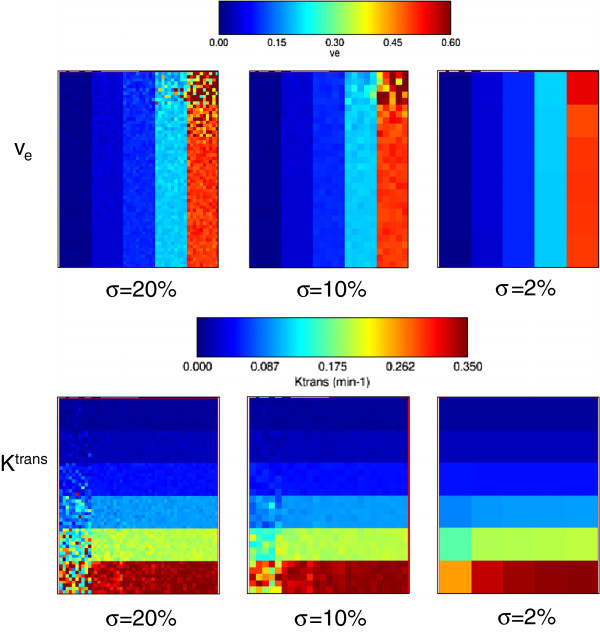
**Parametric map of Tofts model applied to QIBA test data.***K*^*trans*^ and *v*_*e*_ parametric maps calculated over the whole QIBA test data (Tofts model), adding Gaussian noise of *σ*=20% of the signal baseline. Coarser resolutions of 2 ×2 and 10 ×10 pixel size, with an equivalent Gaussian noise of *σ*=10% and 2% are also shown.

**Figure 8:**
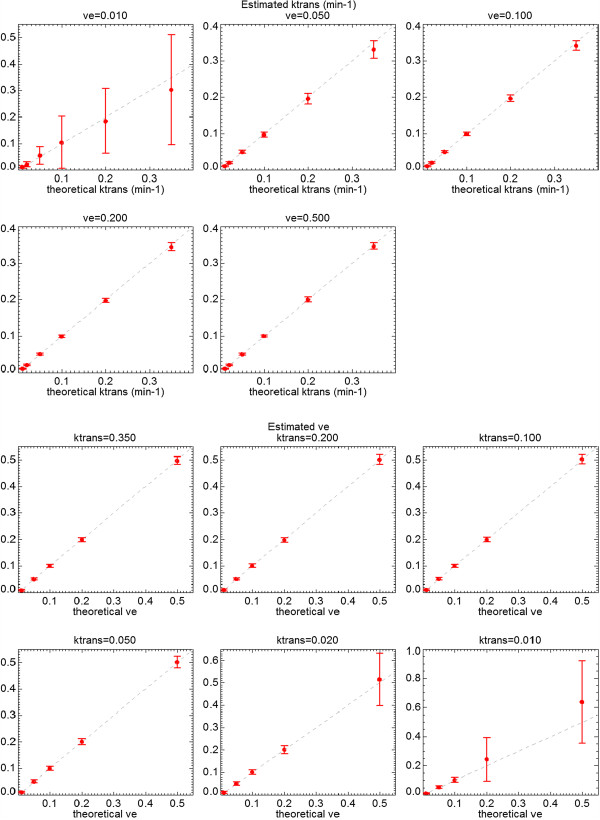
**Tofts model applied to QIBA test data.** Standard deviations of *K*^*trans*^ (upper) and *v*_*e*_ (down) values calculated over the whole QIBA test data (Tofts model), adding Gaussian noise of *σ*=20% of the signal baseline, and compared with the theoretical values plotted as the diagonal line.

Gaussian noise of zero mean and standard deviation (*σ*) equal to 20% of the signal baseline was added to the test data set. Box-type ROI covering the whole tissue region was selected (i.e., 50 ×60 pixels with 30 combinations *K*^*trans*^ and *v*_*e*_ values). Coarser resolutions were also studied (i.e., 2 ×2 and 10 ×10 pixel size), with an equivalent Gaussian noise of *σ*=10% and 2% of the signal baseline, respectively. Noise level of *σ*=20% is appreciated in lower-right images in Figure 5, compared with noise free dynamic values of the same two pixels, shown in the upper-right graphs. The fitting of discrete convolution of Equation 3 was applied to all pixels in the selected ROI. Graphical results for and values are represented in Figure 7. Standard deviations referenced to the theoretical values are represented in Figure 8 for *K*^*trans*^ (up) and *v*_*e*_ (bottom).

#### Results with Extended Tofts model applied to QIBA test data

**Figure 9:**
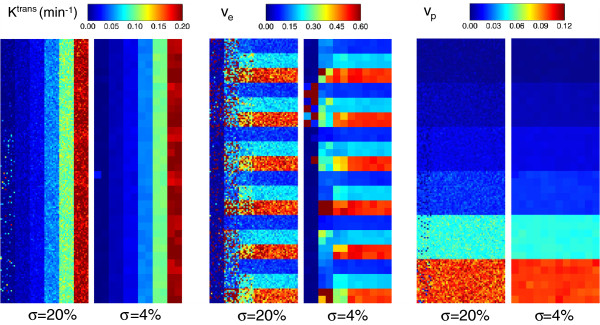
**Parametric map of extended Tofts model applied to QIBA test data.** Values of *K*^*trans*^, *v*_*e*_ and *v*_*p*_ maps using the extended Tofts model supplied in the QIBA test data, adding Gaussian noise of *σ*=20% of the signal baseline. Parametric maps using resolution of 5 ×5 pixels, resulting in an equivalent noise of *σ*=4%, are also shown.

**Figure 10:**
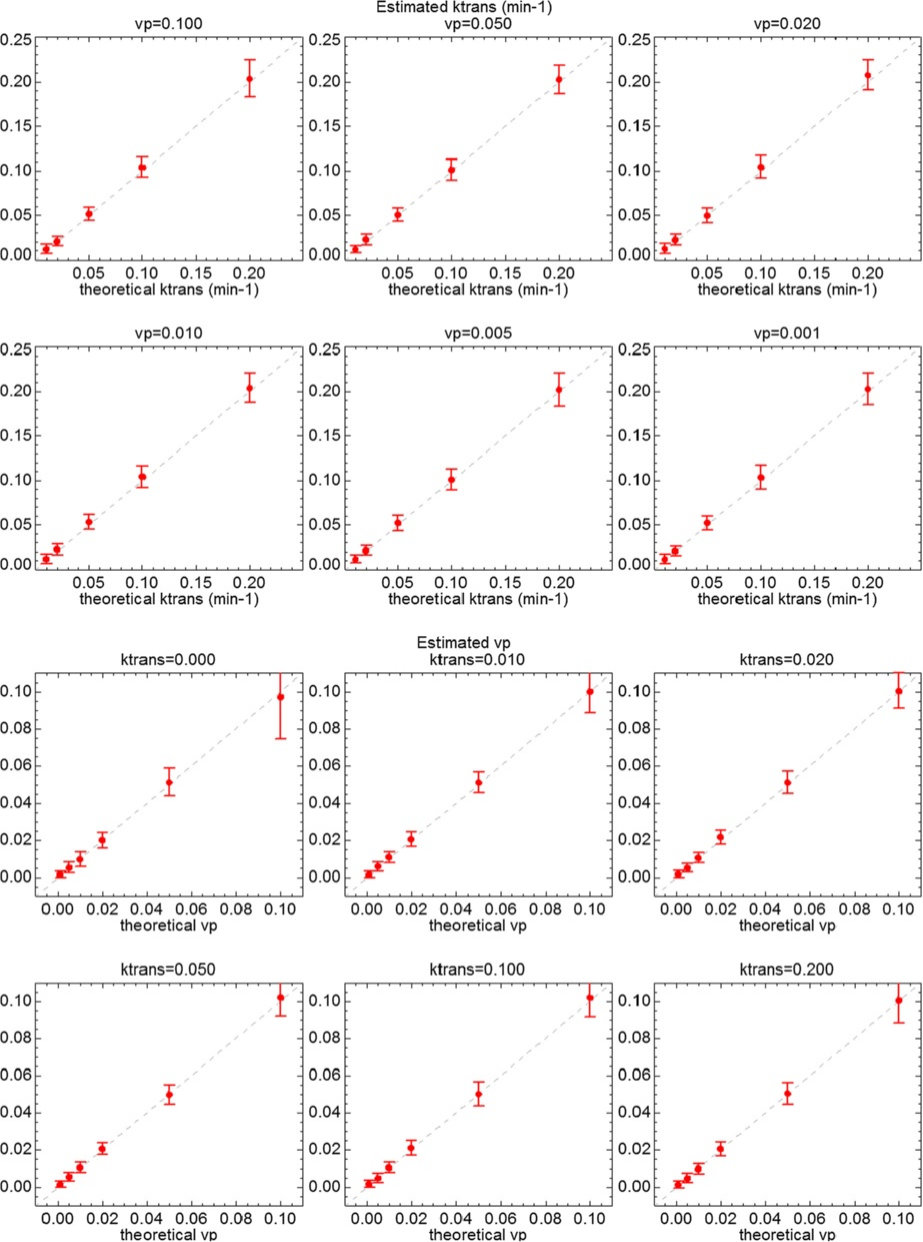
**Extended Tofts model applied to QIBA test data.** Standard deviations of *K*^*trans*^ (upper) and *v*_*p*_ (down) values calculated over the whole QIBA test data of extended Tofts model, adding Gaussian noise of *σ*=20% of the signal baseline. Values are compared with the theoretical values plotted as the diagonal line.

Gaussian noise of zero mean and *σ*=20% of the signal baseline was added to the test data set. A Box-type ROI of 60 ×180 pixels was selected to cover the 108 combinations of *K*^*trans*^, *v*_*e*_ and *v*_*p*_ values. A coarser resolution map of 5 ×5 pixel size, which reduces noise *σ*=4% of the baseline signal level, was also calculated. Color maps of the resultant parameters are represented in Figure 9. Standard deviations and bias referenced to the theoretical values are represented in Figure 10 for *K*^*trans*^ (up) and *v*_*p*_ (bottom). *v*_*e*_=0.5 was used in all cases.

### Example with mouse brain tumor

The platform has been tested over real acquisitions of T_1_-weighted DCE-MRI small animal data. Two different C57BL/6 mouse models have been used in this study. First, a genetically engineered mouse (GEM, S100-v-ErbB; Ink4a-Arf(+/-)), female, age 40 weeks, bearing a Schwannoma (confirmed by histopathological studies carried out by Dr. Martí Pumarola, Murine Pathology Unit, *Centre de Biotecnologia Animal i Teràpia Gènica, UAB*). Animals from this colony generally develop oligodendrogliomas [53], although a small percentage of animals can develop other tumour types [54]. The second model studied was a mouse bearing a stereotactically-induced GL261 glioblastoma, described elsewhere [55,56], age 20 weeks.

A bolus of CA (Gd-DTPA –Magnevist, Bayer Schering Pharma AG, Berlin, Germany–, 50 mM in saline, 0.2 mmol/kg, 10 s duration) was manually injected after acquiring five pre-contrast images. A series of 41 dynamic spin-echo images was acquired with temporal resolution of 51.2 s per frame and the following parameters: TR/TE, 200/5 ms; field of view, 17.6 ×17.6 mm^2^; slice thickness, 1 mm; in-plane resolution, 138 ×138 *μ*m/pixel. The studies were carried out at the joint NMR facility of the Universitat Autònoma de Barcelona and CIBER-BBN (Cerdanyola del Vallès, Spain), using a 7 T horizontal magnet (BioSpec 70/30; Bruker BioSpin, Ettlingen, Germany).

**Figure 11:**
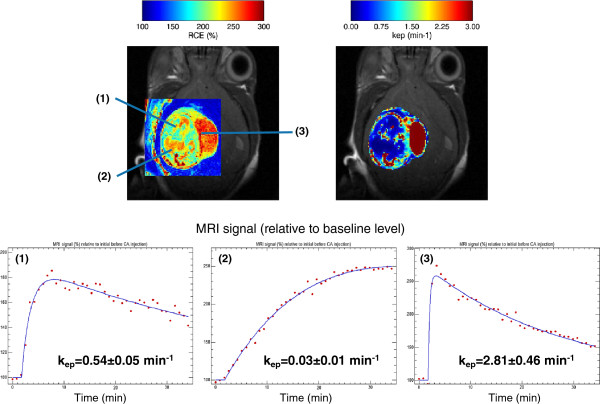
**Pixel-wise Hoffmann analysis over a manually delineated ROI in a mouse Schwannomma.** Upper-left: RCE image over a Box-type ROI with the locations of three pixels. Upper-right: *k*_*ep*_ map over a Free-drawing ROI type (Hoffmann model). Lower: MR signal courses and fitted Hoffmann model in the three selected pixels.

**Figure 12:**
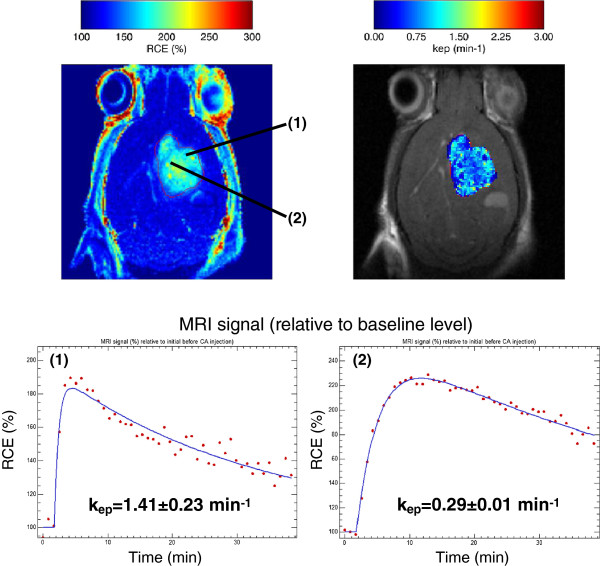
**Pixel-wise Hoffmann analysis over a manually delineated ROI in a stereotactically-induced mouse GL261 glioblastoma.** Upper left: RCE image over a Free-drawing ROI type with the locations of two pixels. Upper right: *k*_*ep*_ map (min ^−1^) over a Free-drawing ROI type (Hoffmann model). Lower: MR signal courses and fitted Hoffmann model in the two selected pixels.

Pixel-wise Hoffmann analyses were performed over a manually delineated ROI in a Z-slice for both cases (Figures 11 and 12, top). The MR signal courses are shown in Figures 11 and 12 (bottom) with significant differences in their biophysical parameters. For the GEM Schwannoma case, *k*_*ep*_ estimated values were 0.54 ±0.05 min ^−1^ for pixel (1) and 0.03 ±0.01 min ^−1^ for pixel (2), while pixel (3) region contains highly vascularised tissue and the Hoffmann model did not apply correctly in these cases (only a few pixels presented acceptable fittings: an example is shown in Figure 11, estimated *k*_*ep*_=2.81±0.46 min^−1^). For the GL261 glioblastoma example (Figure 12), the *k*_*ep*_ estimated values were 1.41 ±0.23 min ^−1^ (tumour border, better perfusion) and 0.29±0.01 min ^−1^ (tumour core). The mean *k*_*ep*_ value for this tumour was 0.77 ±0.35 min ^−1^, which is similar to the mean values calculated for other GL261 cases in our group (0.87±0.59,n=8). These values also agree with previously described studies in the literature. For example, it was possible to calculate *k*_*ep*_ from *K*^*trans*^ and *v*_*e*_ values reported by authors in [46], which studied a rat glioma model: the *k*_*ep*_ value calculated was 0.86 min ^−1^. Regarding to mouse glioma models, the same *k*_*ep*_ estimation approach was possible from the study performed in [7] taking into account graphs in their page 612: the estimated *k*_*ep*_ value in this case was 0.75 min ^−1^, for tumours with a volume (60–80 mm^3^) similar to our GL261 (69 ±43 mm^3^). In both cases, the differences observed in the MR signal time courses between well-perfused and badly perfused (hypoxic regions) agree with the ones described by authors in [15,57].

### Computational implementation and requirements

DCE@urLAB has been implemented in a flexible and modular way, so that the addition of new analysis models is straightforward. The different models can also be used as inline functions to allow flexibility of use and batch programming of multiple studies for advanced users.

Regarding complexity, the optimization (LMA) performed in each pixel has a global algorithmic complexity bound dependent on stopping criterion and number of maximum iterations. The algorithmic complexity by iteration is determined by the cost function (i.e., the pharmacokinetic model) through the calculation of its jacobian matrix. It has been experimentally verified that the computing time needed to perform a pharmacokinetic analysis depends linearly on the number of pixels contained in the ROI and the number of dynamic frames of DCE dataset. This behaviour is expected since the average number of iterations of the LMA does not substantially change for large number of pixels. For example, in a 2.8 GHz Intel Quad Core CPU with 8 GB RAM personal computer, it took 20 seconds to fit a ROI of 1024 pixels and 40 dynamic frames to the Tofts model. Although unrealistic, because tumor ROIs are smaller, the complete analysis using the Tofts model of the whole DCE dynamic slice (128×128=16384 pixels) and 40 dynamic frames, took about 5 minutes in the personal computer formerly described. A maximum of 1.5 GB RAM was required in this case. Should more computer power be required (e.g., with higher resolution images), the program could be easily parallelized and several cores used.

DCE@urLAB is designed to run under Microsoft Windows XP/Vista/7 (both 32 and 64 bits). In order to use the application tool, IDL (version 6.4 or posterior) must have been installed. Another possibility is to install the IDL virtual machine (version 6.4 or posterior), which can be downloaded freely and does not require a license.

## Conclusions

Up to date there is no friendly software application for pixel-wise and ROI analysis of DCE-MRI data that can apply different pharmacokinetic models in a preclinical environment. DCE@urLAB is a user-friendly software designed to fulfill the potential needs of the preclinical DCE-MRI community. It has been focused on the analysis of T_1_-weighted DCE-MRI studies, and tested and optimized according to the requirements of preclinical data analysis. The proposed tool has also been specially designed for easy selection of multi-pixel ROIs. The platform incorporates the compartmental pharmacokinetic models of Tofts, Hoffmann, Larsson, and RR, complemented with non-parametric analysis. Pixel-wise and ROI options allow the user to choose from a variety of forms and pixel sizes (i.e., resolutions). If required by the model, AIF and T_10_ maps can also be estimated from the acquired data. DCE@urLAB reads multi-slice DCE-MRI data from proprietary and binary raw formats. Results can be exported as color maps superimposed to the DCE image, or as text files that can easily be read with other statistical software packages. Individual pixel and ROI dynamic curves can also be visualized, for easy expert interpretation and pharmacokinetics validation. The most relevant and used models in literature (Tofts models) have been validated with publicly available simulated data. Preliminary experiments have been conducted using T_1_-weighted DCE-MRI dynamic data from tumor-bearing mouse brains. A public release of DCE@urLAB, together with the open source code and sample datasets, is available at http://www.die.upm.es/im/archives/DCEurLAB/ and in Additional files 1 and 2.

## Availability and requirements

**Project name:** DCE@urLAB 1.0**Project home page:**http://www.die.upm.es/im/archives/DCEurLAB/**Operating system(s):** Microsoft Windows 7/Vista/XP**Programming language:** IDL**Other requirements:** IDL 6.4 or higher, IDL Virtual Machine 6.4 or higher**License:** BSD license

## Abbreviations

AIF: Arterial input function; BMS: Bulk magnetic susceptibility; CA: Contrast agent; CPU: Central processing unit; DCE: Dynamic contrast-enhanced; DWI: Diffusion weighted imaging; EES: Extracellular extravascular space; Gd-DTPA: Gadolinium-diethylene-triamine penta-acetic acid; GEM: Genetically engineered mouse; GUI: Graphical user interface; IAUC: Initial area under curve; IDL: Interactive Data Language; LMA: Levenberg–Marquardt algorithm; MR: Magnetic resonance; MRI: Magnetic resonance imaging; RAM: Random access memory; RR: Reference region; RCE: Relative contrast enhancement; ROI: Region of Interest; TE: Echo time; TR: Repetitition time; TTM: Time to max enhancement.

## Competing interests

The authors declare that they have no competing interests.

## Authors’ contributions

JEO, RVS, MJLC and APC participated in the design of the application tool. JEO implemented the software. RVS and APC carried out test of the application and software validation MJLC, CA and AS contributed with data interpretation. CA and AS coordinated the work. All authors contributed with the know-how in biomedical imaging, helped to draft the manuscript and read and approved the final version.

## Supplementary Material

Additional file 1 **Compressed file (zip format) with executable software, source code, and user manual.** Unzip and read the file “/help/DCEurLAB_UserGuide.pdf” for instructions and details. Click here for file

Additional file 2 Compressed file (zip format) with examples to test and validate the DCE@urLAB application. Click here for file
