# Supplementary material for: DCE@urLAB: a dynamic contrast-enhanced MRI pharmacokinetic analysis tool for preclinical data
Source: BMC Bioinformatics. 2013 Nov 4;14:316. doi: 10.1186/1471-2105-14-316 (PMC4228420; doi:10.1186/1471-2105-14-316)
Supplement: Additional file 1 — Compressed file (zip format) with executable software, source code, and user manual. Unzip and read the file “/help/DCEurLAB_UserGuide.pdf” for instructions and details. [file 1471-2105-14-316-S1.zip › Help/DCEurLAB_Methods.pdf]

DCE@urLAB methods

This is a summary of the methodology used in DCE@urLAB application

Introduction:

DCE-MRI (Dynamic Contrast Enhancement Magnetic Resonance Imaging) is a biomedical modality that involves the serial acquisition of MR images of a tissue of interest before and after an intravenous injection of an exogenous contrast agent (CA). As the CA enters into the tissue, the longitudinal (T_1) and transversal (T_2) relaxation times of the MR are modified to an extent that is determined by the concentration of the agent. By considering a set of images acquired before and after the CA injection, a region of interest (ROI) or individual pixels will exhibit a characteristic signal intensity time course, which can be related to CA concentration. By fitting the DCE-MRI data to an appropriate pharmacokinetic (PK) model, physiological and quantitative parameters can be extracted (Tofts and Kermode, 1991).

General Assumptions

PK models includes in DCE-MRI assume general concepts in tracer kinetics and magnetic resonance (Tofts, 1997):

- There exist certain compartments that contain a well-mixed CA with uniform concentration.
- The flux between compartments is directly proportional to the difference of concentration of CA between the two compartments (i.e., linear inter-compartment flux).
- Parameters describing the compartments don't' change during the experiment (i.e., time invariance).
- The increase in longitudinal relaxation rate (i.e., the inverse of T_1) is proportional to the concentration of CA by a constant factor, denoted as longitudinal relaxivity.

Compartments

PK models applied have two compartments in common: the blood plasma compartment and the Extracellular-extra vascular (EES) compartment. In the brain, the two compartments are separated by the blood-brain barrier (BBB). As the CA doesn't enter in the intracellular space, so it is not considered in the PK models. The blood plasma compartment is the central compartment, with wash-out to the kidneys and intake from the injected contrast, while the EES compartment is the peripheral compartment. This scheme is pointed in figure 1.

In DCE-MRI PK analysis, changes of concentration of CA in tissue over time are modeled as a result of first-order exchange of CA molecules between compartments. The general mathematical form of the PK model is independent of the CA exchange biological mechanism (i.e., blood flow, permeability, or a mixed case). The generalized PK model is expressed as a modified general rate equation (S. S. Kety 1951),(Tofts*, et al.*, 1999), which describes the rate of accumulation and wash-out of CA in the EES:

$$\frac{dC_e(t)}{dt} = \frac{K^{trans}}{v_e}\left(C_P(t) - C_e(t)\right)$$
$$v_p C_P(t) + v_e C_e(t) = C_t(t) \qquad (1)$$

where C_p is the CA concentration in plasma, C_e is the CA concentration in EES, v_e is the fractional volume of EES, and K^{trans} is the volume transfer constant between plasma and EES. Equation 1 can be expressed also as function of the rate constant between EES and plasma (k_{ep}), which is related through $k_{ep} = K^{trans} / v_e$

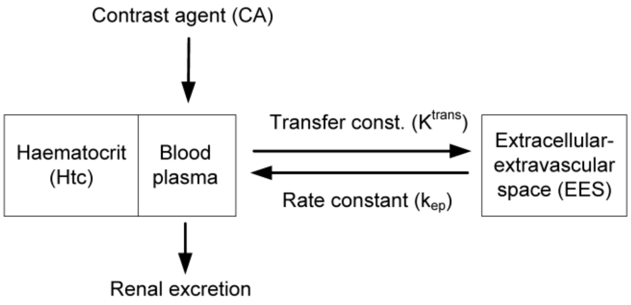

Figure 1: Two-compartmental model accessed by low molecular weight CA

Biological interpretation of K^{trans}

If the delivery of CA to the tissue is ample (i.e., the rate of extraction of CA via the BBB is small compared with the rate of replenishment by perfusion), K^{trans} is equivalent to the product of capillary wall permeability and capillary wall surface area per unit volume. This is usually expressed as $K^{trans} = PS\rho$, where PS is the permeability surface area product per unit of mass of tissue (ml $g^{-1}min^{-1}$) and ρ is the tissue density (g ml^{-1}). This situation is commonly named as "flow limited"

On the other hand, if the delivery of CA is insufficient, blood perfusion will be the dominant factor determining the CA kinetics, and the behavior of the tissue it will be "permeability limited" with $PS \ll F$. In this case, $K^{trans} = F\rho(1 - Hct)$, where F is the flow or perfusion (ml $g^{-1}min^{-1}$) and Hct is the hematocrit.

In a mixed situation, not limited by flow or by permeability, $K^{trans} = E F \rho(1 - Hct)$, where E is the CA extraction fraction, expressed as:

$$E = 1 - \exp\left(\frac{PS}{F(1 - Hct)}\right) \qquad (2)$$

Tofts model

The Tofts model (Tofts and Kermode, 1991) derives from the generalized bi-compartmental PK model of equation 1. It assumes that the contrast agent is injected as a bolus and instantaneously is distributed in

the vascular plasma compartment. The general solution to the first order differential equation (1) with initial conditions $C_p(0)=C_e(0)=0$ is:

$$C_t(t)=K^{trans}\int_0^t C_p(t')e^{\left(-K^{trans}(t-t')/v_e\right)}dt' \tag{3}$$

C_p is modeled with a bi-exponential decay in the original Tofts model {Tofts, 1991 #17}:

$$C_p(t)=D\sum_{i=1}^{2}a_i\exp(m_i t) \tag{4}$$

where D is the dose (mmole/kg), amplitudes a_i are normalized for unit dose in kg/litre, and rate constant m_i have units of min^{-1}. Using Equation 4 to solve Equation 2:

$$C_t(t)=DK^{trans}\sum_{i=1}^{2}a_i\left(\frac{\exp(-k_{ep}t)-\exp(-m_i t)}{m_i-k_{ep}}\right) \tag{5}$$

where unknowns parameters are K^{trans} and k_{ep}. This original Tofts model do not take into account any significant vascular signal, so $C_t(t)=v_eC_e(t)$. If the effects of the plasma compartment to the total signal is not negligible, (i.e., $C_t(t)=v_eC_e(t)+v_pC_p(t)$), and additional term is added to equation 4, leading to the modified or extended Tofts model, with the additional unknown parameter v_p:

$$C_t(t)=DK^{trans}\sum_{i=1}^{2}a_i\left(\frac{\exp(-k_{ep}t)-\exp(-m_i t)}{m_i-k_{ep}}\right)+v_pD\sum_{i=1}^{2}a_i\exp(m_i t) \tag{6}$$

Tofts model can be interpreted as a convolution with a residue function $H(t)=K^{trans}e^{-k_{ep}t}$:

$$C_t(t)=C_p(t)*H(t) \tag{7}$$

where the symbol * is the convolution operator. The vascular component is added in the modified Tofts model:

$$C_t(t)=C_p(t)*H(t)+v_pC_p(t) \tag{8}$$

The discrete approximation of equation 7 considers that $C_t(t)$ and $C_p(t)$ are measured at a set of equally spaced time points $t_1,t_2,...,t_N$; $t_{i+1}-t_i=\Delta t$ and over small time intervals $H(t)$ and $C_p(t)$ are assumed constants:

$$C_t(t_j)\simeq\Delta t\sum_{i=0}^{j}C_p(t_i)H(t_j-t_i) \tag{9}$$

As a linear operator, the discrete convolution can be expressed in a matrix form:

$$\begin{bmatrix} C_t(t_1) \\ C_t(t_2) \\ \vdots \\ C_t(t_N) \end{bmatrix} = \Delta t \begin{bmatrix} C_p(t_1) & 0 & \cdots & 0 \\ C_p(t_2) & C_p(t_1) & \ldots & 0 \\ \vdots & \cdots & \ddots & \vdots \\ C_p(t_N) & C_p(t_N - 1) & \cdots & C_p(t_1) \end{bmatrix} \cdot \begin{bmatrix} H(t_1) \\ H(t_2) \\ \vdots \\ H(t_N) \end{bmatrix} \tag{10}$$

which is solved iteratively with least-squares minimization methods.

Tofts model not should be applied without a T_{10} map and a reliable adjust of C_p to the bi-exponential function. If TR >> T_1, MR signal values are reasonable linear with respect to C_t except for an unknown constant, and the PK model can be useful for semi-quantitative measurements. In this case units K^{trans} are not related to C_t, and even can give results greater than the unit.

Note that C_t must be calibrated from the MRI data. With T1-weighted DCE-MRI studies, the process is divided detailed in the MRI model section.

Hoffmann model

It consist of a two-compartment model proposed by (Hoffmann*, et al.*, 1995) based on that of (Brix*, et al.*, 1991), which incorporates rate constants of CA between the lesion to plasma compartments(k_{ep}), and the clearance rate (k_{el}). The plasma concentration not need to be directly measured because k_{el} can be estimated from the measured tissue curve. After a bolus injection of duration τ, if It is assumed that $k_{ep}\tau << 1$ and $k_{el}\tau << 1$. CA transfer between blood plasma to EES is supposed to be a slow process. i.e., $k_{ep}, k_{el} >> k_{pe}$. Hoffmann model reduces to equation 11 (Tofts, 1997):

$$\frac{S(t)}{S_0} = 1 + A^H k_{ep} \frac{\exp(-k_{ep}t) - \exp(-k_{el}t)}{k_{el} - k_{ep}} \tag{11}$$

where $S(t)$ is the MR signal of the sequence from tissue and S_0 is the initial signal before CA injection. The fitting parameters are: A^H (normalized amplitude, which approximately corresponds to the size of the EES), k_{ep} and k_{el}. The model deals with the situation in which the arterial concentration is unknown, and C_p cannot be correctly estimated. Instead, the blood compartment is assumed to have a constant k_{el}. C_p is assumed to be the result of a constant infusion of CA with known duration. Also tissues have a negligible vascular component in this model.

At short times after injection, the right side of (9) reduces to $1 + Ak_{ep}$; thus, the initial slope of the curve is proportional to Ak_{ep}.

Larsson model

Larsson model (Larsson*, et al.*, 1990) simplifies the Tofts model assuming a known blood plasma CA course, either measured from blood samples or estimated from MRI. This model is mono-compartmental. The application tool assumes that the MR signal is linearly related to the CA concentration. In that case, the signal is modeled as:

$$S(t) = S_0 + \frac{\dot{S}(t)}{\sum_{i=1}^{N} a_i} \sum_{i=1}^{N} \frac{a_i \left(\exp\left(-K^{trans} t / v_e\right) - \exp\left(-m_i t\right) \right)}{m_i - K^{trans} / v_e} \tag{10}$$

where $\dot{S}(t)$ is the initial slope of the MR signal, and C_p is modeled as a sum of N exponentials with amplitudes a_i and time constant m_i. Although the original model proposed three exponentials (N=3) DCE@URLAB implements a two-exponential model for C_p (as in Tofts model).

Reference region model

An alternative to a populations based or estimated is the reference region (RR) approach {Yankeelov, 2005 #256}. The modeling by the RR approach uses a well-characterized tissue to combine two copies of equation (1), one for the tumor and other for the reference region:

$$\frac{dC_{t-RR}(t)}{dt} = K^{trans-RR} C_p(t) - \frac{K^{trans-RR}}{v_{e-RR}} C_{t-RR}(t) \tag{11}$$

where C_{t-RR} is the concentration of CC in the RR tissue and $K^{trans-RR}$ and $v_{e,RR}$ are the quantitative parameters for the RR. The pair of equations allows for elimination of C_p, and the solution is given by:

$$C_t(t) = \frac{K^{trans}}{K^{trans-RR}} \left(C_{t-RR}(t) + \left(\frac{K^{trans-RR}}{v_{e-RR}} - \frac{K^{trans}}{v_e} \right) \int_0^t C_{t-RR}(t') e^{-\left(\frac{K^{trans}}{v_e}\right)(t-t')} dt' \right) \tag{12}$$

MRI model

Tofts and RR models require the calibration of CA concentration from measured MRI parameters. If the bulk magnetic susceptibility (BMS) shift is negligible, the relationship between T_1 and CA concentration is determined by the Solomon-Bloembergen equation (Haase et al. 1986).

$$\frac{1}{T_1(t)} = \frac{1}{T_{10}} + r_1 C_t(t) \tag{13}$$

where T_{10} is the T_1 value before CA injection and r_1 is the longitudinal relaxivity (assumed constant).The relationship between CA concentration and the relative increase in signal intensity can be derived from the Bloch equations for any imaging sequence, e. g., the signal for a T_1-weighted spin-echo pulse sequence (at short echo time) with repetition time (*TR*) is:

$$S(t) = S_0 \left(1 - \exp\left(-\frac{TR}{T_1(t)} \right) \right) \tag{14}$$

Where S_0 is the MR signal prior to CA injection and TR is the MR repetition time. From Equations 13) and known values of T_{10} prior to the CA injection it is possible to calibrate $T_1(t)$.

Equations (13) and (14) are joined in the equation 15 that gives tissue concentration from MR signal:

$$C_t(t)=\frac{-\ln\left(1-\frac{S(t)}{S_o}\left(1-\exp\left(-\frac{TR}{T_{10}}\right)\right)\right)-\frac{TR}{T_{10}}}{r_1 TR} \tag{15}$$

For spoiled gradient-echo pulse sequences with flip angle α, MR signal is equal to:

$$S(t)=\frac{S_0\left(1-e^{-TR/T_1(t)}\right)\sin(\alpha)}{1-\cos(\alpha)e^{-TR/T_1(t)}} \tag{16}$$

Signal intensity is converted to CA concentration in tissue using the equation from (Li et al. 2000)) to get the longitudinal relaxation rate $R_1(t)$:

$$R_1(t)=-\left(\frac{1}{TR}\right)\ln\left(\frac{1-\left(\frac{S(t)-S(0)}{S_0\sin(\alpha)}+\frac{1-m}{1-m\cos(\alpha)}\right)}{1-\cos(\alpha)\left(\frac{S(t)-S(0)}{S_0\sin(\alpha)}+\frac{1-m}{1-m\cos(\alpha)}\right)}\right),\quad m=e^{-TR/T_1} \tag{16}$$

and CA concentration is calculated as $C(t)=\left(R_1(t)-R_1(0)\right)/r_1$. Note that r_1 and T_{10} must be known to quantify the tissue concentration from the MR signal. T_{10} may be estimated using the ratio of two spin-echo images collected with different TR. Estimation error can be reduced with a higher number of images with a least-squares minimization algorithm.

Curve fitting

uses the Levenberg-Marquardt algorithm (LMA) (Marquardt, 1963) to perform a least squares curve fitting of equations (4),(5),(9) and (10). LMA has demonstrated to be robust in pharmacokinetic modeling of DCE-MRI (Ahearn*, et al.*, 2005).

References

T. S. Ahearn, R. T. Staff, T. W. Redpath, and S. I. K. Semple, “The use of the Levenberg-Marquardt curve-fitting algorithm in pharmacokinetic modelling of DCE-MRI data”, *Physics in Medicine and Biology,* vol. 50, no. 9, pp. N85-N92, May 7, 2005.

G. Brix, W. Semmler, R. Port, L. R. Schad, G. Layer *et al.*, “Pharmacokinetic Parameters in CNS Gd-DTPA Enhanced Mr Imaging”, *Journal of Computer Assisted Tomography,* vol. 15, no. 4, pp. 621-628, Jul-Aug, 1991.

U. Hoffmann, G. Brix, M. V. Knopp, T. Hess, and W. J. Lorenz, “Pharmacokinetic Mapping of the Breast - a New Method for Dynamic Mr Mammography”, *Magnetic Resonance in Medicine,* vol. 33, no. 4, pp. 506-514, Apr, 1995.

H. B. W. Larsson, M. Stubgaard, J. L. Frederiksen, M. Jensen, O. Henriksen *et al.*, “Quantitation of Blood-Brain-Barrier Defect by Magnetic-Resonance-Imaging and Gadolinium-Dtpa in Patients with Multiple-Sclerosis and Brain-Tumors”, *Magnetic Resonance in Medicine,* vol. 16, no. 1, pp. 117-131, Oct, 1990.

D. W. Marquardt, “An Algorithm for Least-Squares Estimation of Nonlinear Parameters”, *Journal of the Society for Industrial and Applied Mathematics,* vol. 11, no. 2, pp. 431-441, 1963.

P. S. Tofts, “Modeling tracer kinetics in dynamic Gd-DTPA MR imaging”, *Jmri-Journal of Magnetic Resonance Imaging,* vol. 7, no. 1, pp. 91-101, Jan-Feb, 1997.

P. S. Tofts, G. Brix, D. L. Buckley, J. L. Evelhoch, E. Henderson *et al.*, “Estimating kinetic parameters from dynamic contrast-enhanced T-1-weighted MRI of a diffusable tracer: Standardized quantities and symbols”, *Journal of Magnetic Resonance Imaging,* vol. 10, no. 3, pp. 223-232, Sep, 1999.

P. S. Tofts, and A. G. Kermode, “Measurement of the Blood-Brain-Barrier Permeability and Leakage Space Using Dynamic Mr Imaging .1. Fundamental-Concepts”, *Magnetic Resonance in Medicine,* vol. 17, no. 2, pp. 357-367, Feb, 1991.

Corresponding author:

Juan Enrique Ortuño
Biomedical Image Technologies (BIT)
Dpto. de Ingeniería Electrónica, Universidad Politécnica de Madrid (UPM)
Av. Complutense "Ciudad Universitaria" nº 30, E-28040 Madrid (Spain)
e-mail: juanen@die.upm.es
web : www.die.upm.es/im
