# Supplementary material for: DCE@urLAB: a dynamic contrast-enhanced MRI pharmacokinetic analysis tool for preclinical data
Source: BMC Bioinformatics. 2013 Nov 4;14:316. doi: 10.1186/1471-2105-14-316 (PMC4228420; doi:10.1186/1471-2105-14-316)
Supplement: Additional file 1 — Compressed file (zip format) with executable software, source code, and user manual. Unzip and read the file “/help/DCEurLAB_UserGuide.pdf” for instructions and details. [file 1471-2105-14-316-S1.zip › Help/DCEurLAB_UserGuide.pdf]

# DCE@urLAB user guide

06-06-2013 BIT-UPM

## Introduction:

**DCE@urLAB** is a software application for analysis of dynamic T1-weighted perfusion MR images. The user can manually select ROIs (regions of interest) for applying different pharmacokinetic models. Currently, Tofts model, Brix/Hoffmann and Larsson model have been implemented. For a detailed description of these methods please consult the "DCEurLAB_*methods.pdf*" document.

## System requirements:

### *Software:*

DCE@urLAB is designed to run under operating system Microsoft Windows XP/Vista/7 (32 and 64 bits). DCE@urLAB has been developed under IDL (©Exelis Visual Information Solutions). In order to use this software, you must have IDL installed on your system (version 6.4 or posterior), or can use the IDL virtual machine, which can be used without a license. IDL virtual machine can be downloaded from http://www.exelisvis.com/idlvm/

### *Hardware:*

Processing of dynamic T1-weighted DCE-MRI data can be a processor and memory intensive task. The memory and CPU resources needed to process the dynamic images vary depending on the size of the data sets involved. For large data sets, above 2 GB of RAM memory is recommended. In the help menu, there is a memory requirements button than tells the maximum memory needed to process the data currently loaded in the application.

## Installation:

Simply decompress the RAR file in a directory. It contains the following files and directories:

- **DECurLAB.sav**. The main binary file that must be loaded with IDL or IDL virtual machine.
- **DCEurLAB_config.txt** Configuration file that contains the parameters loaded when the application is executed.
- **Icons** directory with auxiliary files.

These filenames (included the auxiliary ones) must not be changed or the software might not work properly

- **Config** directory, with alternative configuration files
- **Source** directory, which contains the IDL source code

- **Help** directory, which contains the user guide and the description of methods.
- **Examples** directory, with DCE-MRI data and T1 maps

## Using DCE@urLAB:

You must already IDL (version 6.4 or posterior) or IDL virtual machine installed in your computer. To execute **DCE@urLAB**, simply open a window explorer and press the **DECurLAB.sav** file. If IDL or IDL virtual machine is assigned to the “sav” extension, the application will be launched. If another application has been associated to the “sav” extension, you should select IDL or IDL virtual machine manually with the right button of the mouse under the option “Open with → choose program”.

If an IDL license is present, the application will run in a licensed copy of IDL. If no license is present, the IDL Virtual Machine window will open; click anywhere in the window to run the application in the IDL Virtual Machine.

To launch the application, the disclaimer must be accepted (Figure 1)

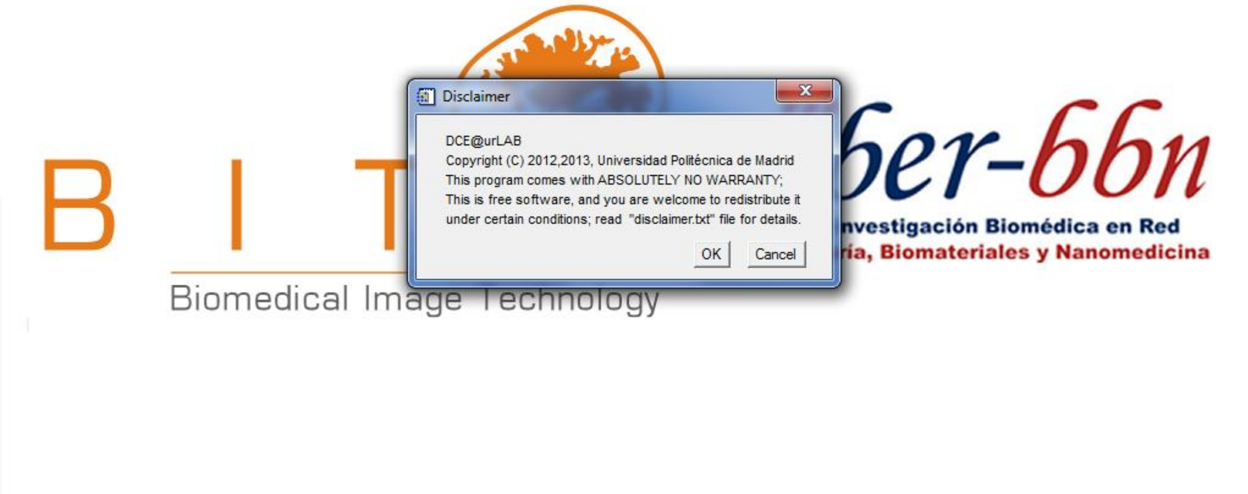


**Figure 1:** the "Welcome" window will appear when DCE@urLAB is executed.

### Executing from the IDL development environment

If the user has a IDL license, the application will can be alternatively launched from the source code included in this distribution. The user has to execute the following commands from the IDL command line:

```
CD, 'E:\DCE-urLAB\Source' ;Change the working directory to your actual source directory;
RESOLVE_ROUTINE, ['mpfit','mpfitfun','hcolorbar2__define','main'], /COMPILE_FULL_FILE, /EITHER;
RESOLVE_ALL, /CONTINUE_ON_ERROR  ;Compile all dependant functions
MAIN ; Execute the main function
```

## Initial parameters

When the application starts, loads the configuration stored in the file *DCEurLAB_config.txt*. The user can change the original values to their preferences. The options and parameters are described in the *Configuration file* section.

If the configuration file is missing or the name has changed, the application asks the user to load an alternative file. This option leaves the user the possibility to quickly change the basic settings between executions.

If the user finally don't choose a configuration file or loads a not valid one, the application ask if the user wants to load the internal default configuration or exit (Figure 3)

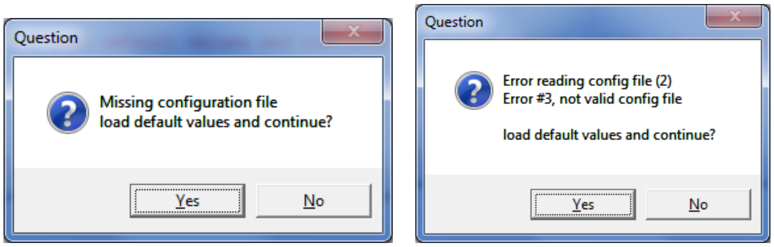


**Figure 2.** Error messages when configuration file is missing or it contains a format error

When the settings are correctly read, a graphical interface similar to Figure 3 will open[1]:

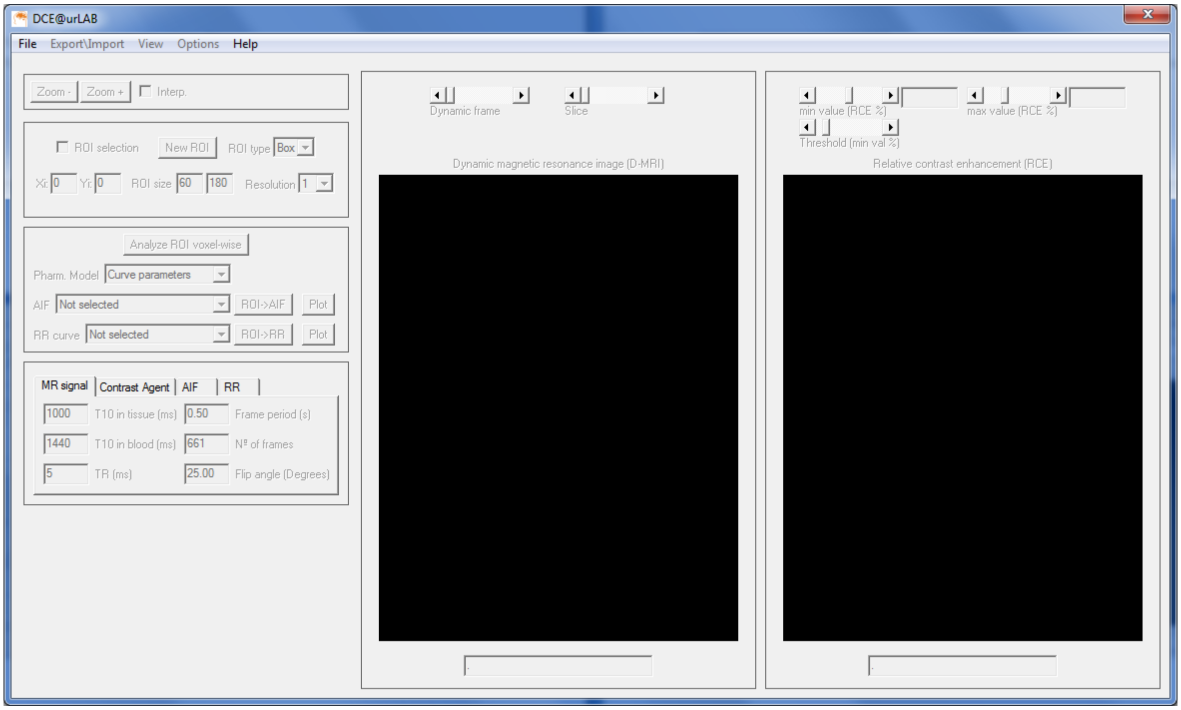


**Figure 3:** Main graphical interface (GUI)

[1] The visual aspect of the interface in this document corresponds to Windows 7 using the Windows Classic Theme. Text size Colors and general aspect may vary if visual environment changes.

# Load MRI data

Graphical Interface (GUI) functionality is disabled until any valid DCE-MRI acquisition is loaded. Allowed formats are included in the main menu, under the button `[file-►Open DCE-MRI]` (Figure 4)

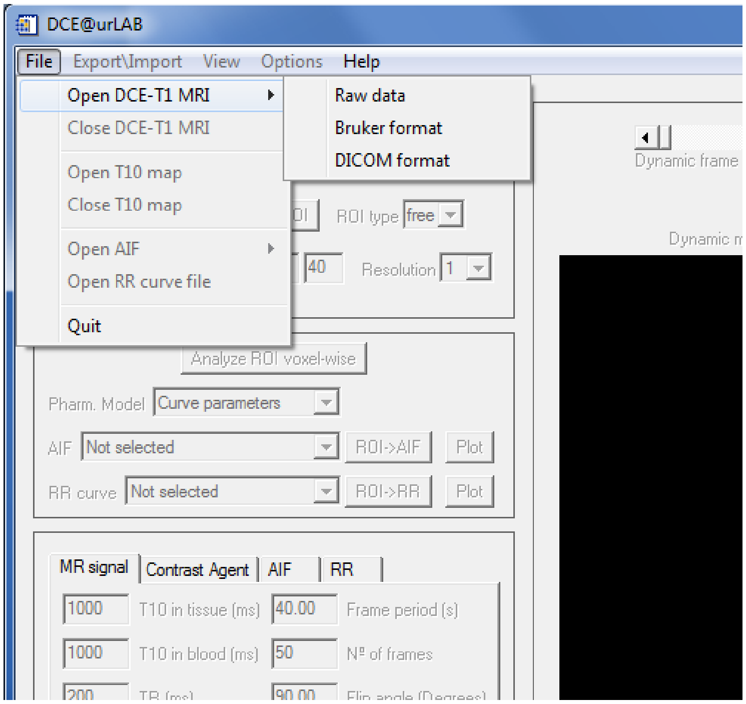


**Figure 4:** Main GUI with file menu opened showing the DCE-MRI formats allowed

This application works with data from dynamic T1-weighted perfusion sequences. The software considers the image set to be a four dimensional stack of images in x-y-z-t order, where x-y is the transaxial plane, z is the axial-axis direction, and t is the frame time. Data can be imported from a binary file as well in Bruker Biospin proprietary format and DICOM format. Data conversion from another format (e.g., Analyze or Nifty) must be performed by the user before using **DCE@urLAB**.

### *Load Bruker file format*

This type of input is selected with the option `[file-►DCE-MRI-►Bruker Format]`. Bruker data are organized in a set of directories. The dialog message asks to load a folder. The user must select the folder containing a file name <2dseq> (without extension). This file contains the binary information. The selected folder must also contain the header named <visu_pars> or <reco>. Additionally, a header named <method> it will be located in the parent or (parent of parent) directory.

If information read in the two headers is not consistent with the binary file or it not contains any dynamic MRI data, a lecture error warning will appear. The user must ensure than the read images are true DCE-MRI data and not another type of acquisition.

Bruker format not include information about the injection frame. So, an additional widget appears immediately after opening the file. The user must enter the injection frame number. The first frame is numbered frame 1. This setting can be changed after the data is loaded.

### *Load unformatted binary files*

This format is selected with the option [file-►Open DCE-MRI-►Raw data]. When this option is pressed, a dialog is opened to choose the file, and after that, an auxiliary widget appears to choose dimensions, coordinate order and format (Figure 5)

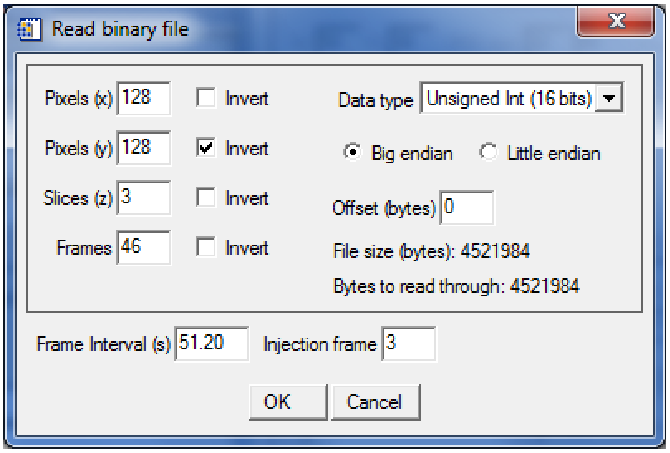


**Figure 5:** Interface for reading binary format data

Data pixel dimensions in x,y,z dimensions and time (t) are selected with the fields [pixels(x)], [pixels(y)], [Slices(z)] and [Frames]. Binary data must be stored in x-y-z-t order (from less to more significant dimensions) to be read properly, because this package version cannot apply an arbitrary transform to the imaging data into (x, y, z, t) space. 4D data can be transposed in a chosen dimension selecting the option [invert].

The proper binary data type is selected in the drop list [data type]. Included options are byte (8 bits), integer of 16, 32 and 64 bits with and without sign, and floating format of 32 and 64 bits. Big-endian and little-endian options can be selected with the exclusive buttons located below the drop list of type format.

To allow the lecture of certain formats with binary data that includes headers, an offset field [Offset (bytes)] has been included. Data will be read from the chosen byte marked in this option.

If there are no additional headers after the last binary data, the raw file size should coincide with the dimensions and data type introduced. The coincidence can be checked if the [file size (bytes)] label matches with the [Bytes to read through] label.

The raw format loading interface also contains the field [frame interval (s)] and [injection frame] to select the period of time between dynamic frames, in seconds, and the frame when contrast is injected. This information can be changed after the data is loaded.

### *Load DICOM file format*

DICOM (Digital Imaging and Communications in Medicine) file format DCM files are opened choosing the proper folder. **DCE@urLAB** must be executed using a 32 bits IDL version to be able to open DICOM format.

As it happens with Bruker format, a floating widget asks for the frame time and injection frame, if this information is not available in the DICOM header.

## *Displaying DCE-MRI images*

If dynamic DCE-MRI data is loaded properly, the interface will show a single 2D slice of the whole 4D data set in the left window, and a RCE (relative contrast enhancement) image in the right window, as it is shown in the example of the Figure 6.

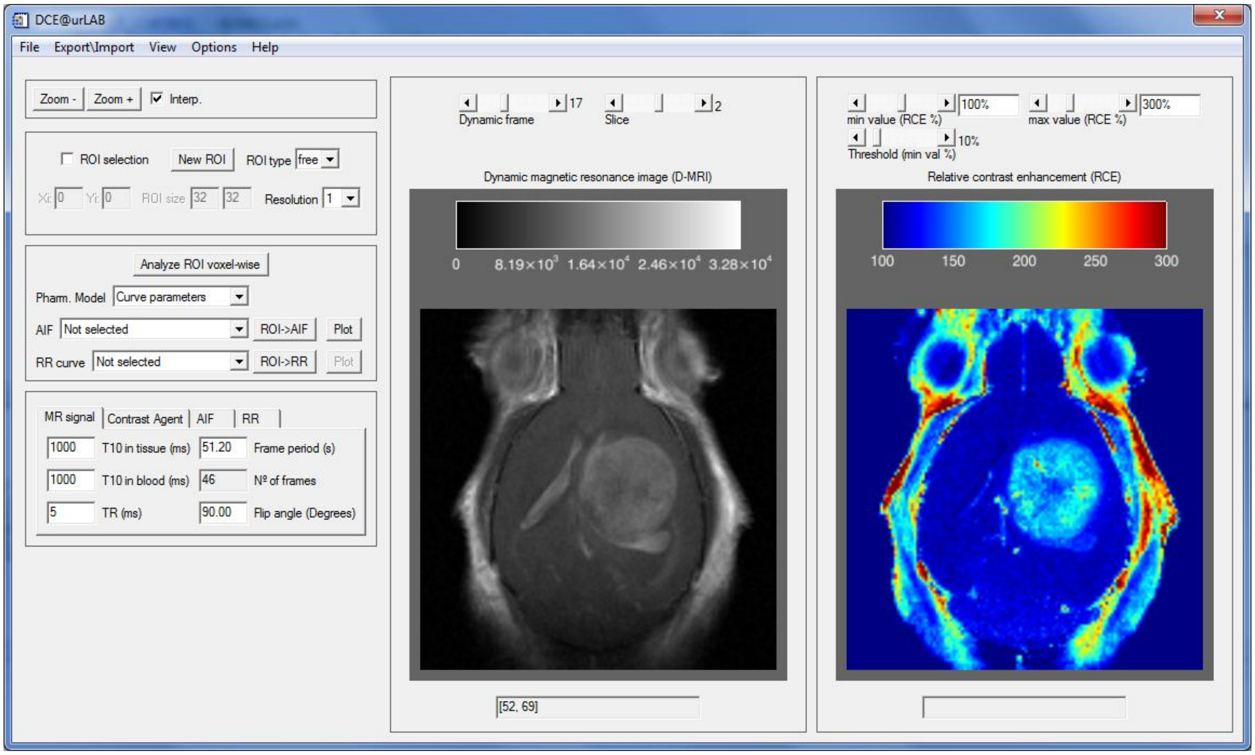


**Figure 6:** Main graphical interface after loading DCE-MRI data

The RCE window can be hidden unselecting the option [View–►View RCE image] in the menu bar. Zoom buttons [Zoom +] and [Zoom-] are used to zoom in and out on the working display area. User can turn interpolation on and off using by selecting the [interp] check-box in the upper-left panel.

The temporal frame and axial slice displayed in the left window is controlled by the [dynamic frame] and [slice] sliders, respectively.

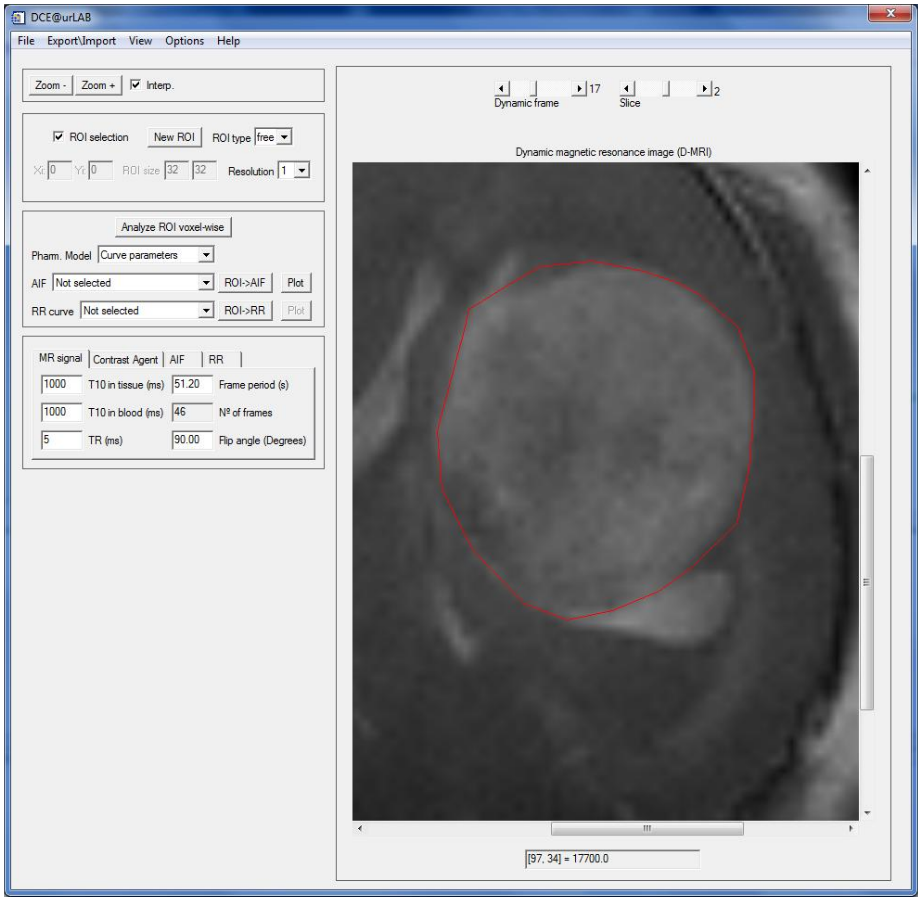


**Figure 7**. Zooming the region of interest of image displayed in Figure 6.

### *Load T1-maps*

Some pharmacokinetic models (e.g., Tofts and RR models integrated in the application) also require a T10 map of the same spatial dimensions of loaded T1-weighted DCE-MRI data. This option is selected in `[File–►open T10 map]`. As the loading of T10-maps is allowed only after the DCE-MRI data has been loaded, dimensions must match in (x,y,z). When dimensions do not match, the application asks the user to perform a linear interpolation. The file format must be floating point and little-endian and unformatted binary. The loaded image is displayed with the option `[View–►View T10 map]` in the menu bar

## Viewing data sets and selecting ROIs

After loading a valid DCE-MRI sequence, processing options and menu bar options becomes activated. The user is now able to select ROIs, parameters, as change initial visualization settings.

### *Choosing dynamic frames and slices*

DCE-MRI sets are four-dimensional data. The slide `[Dynamic frame]` allow the user to select the dynamic frame which is visualized in the interface, while the slide `[Slice]` is used to change between

different slices of the same dynamic frame, but different Z dimension. The RCE image map also changes according with this slide.

### *Image color map*

Color maps of both DCE-MRI image and RCE image can be modified in the menu option [Options-►Color Palette-►MRI] and [Options-►Color Palette-►RCE] .The auxiliary widget is shown in Figure 8. The user can choose between 40 predefined tables as well as modify it.

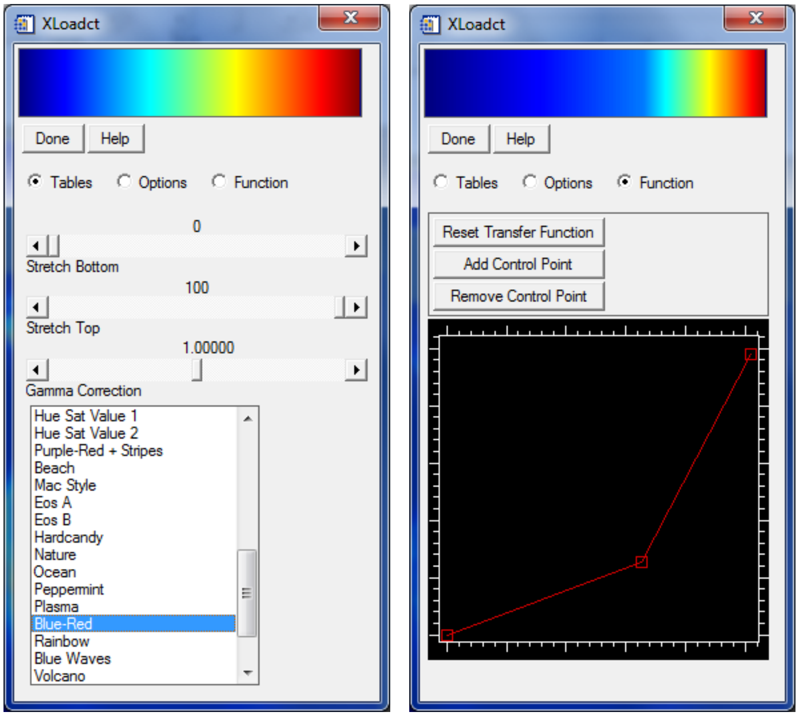


**Figure 8**. Widget to choose the color map applied to the displayed images.

The MR data loads the “black-and-white” table by default, while the RCE image uses the blue-red table. In addition to this, RCE image displayed in the right window has fast controls, [min value (RCE %)] and [max value (RCE %)], to limit the RCE value between a minimum and a maximum, expressed as a percentage. Furthermore, to avoid a *salty* or *random* effect in the background where signal is too noisy relative to the first frames, a minimum threshold is established with the [threshold (min value)].

### *ROI selection mode not activated*

If the check box [ROI selection] is not activated, pressing the right mouse button over any image will plot the dynamic or kinetic curve of the current voxel (Figure 9) as a percentage relative to the mean value before injection of contrast agent.

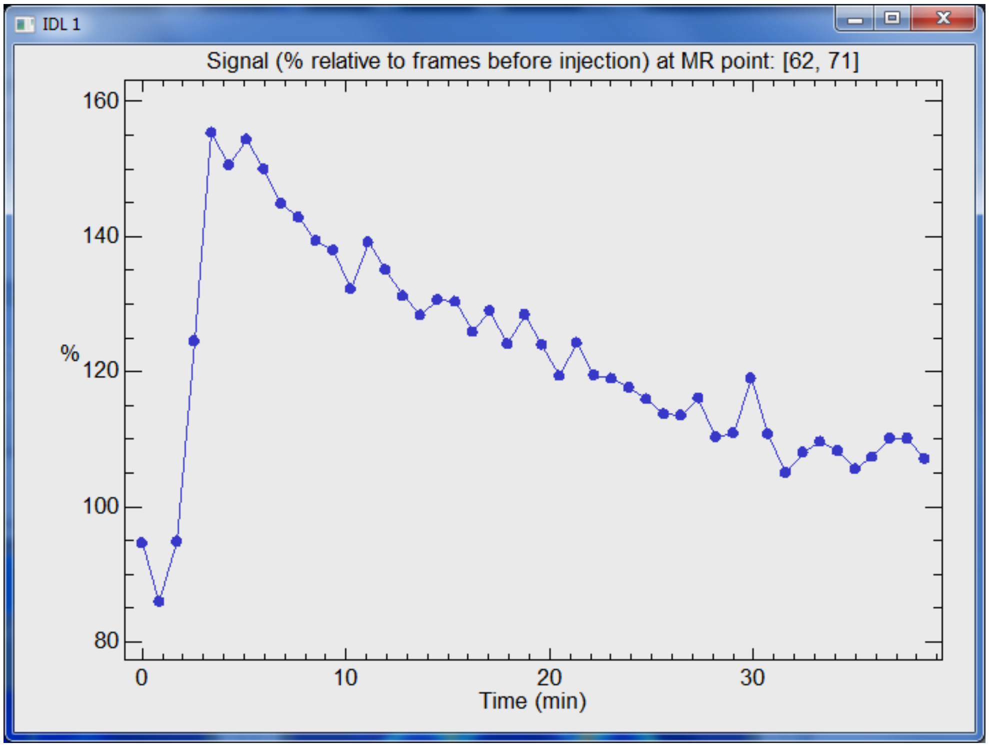


**Figure 9.** Dynamic curve of a single voxel when the right mouse button is pressed.

If the left mouse button is pressed over the DCE-MRI image, the value of the current voxel will appear in the associated information label, located below the display image.

When the left mouse button is pressed over the RCE image, the RCE value of the current voxel (in %) will we shown in the associated information label.

### *ROI selection mode activated*

The ROI selection options are grouped in the left side of the main graphical interface. The detail is shown in Figure 10:

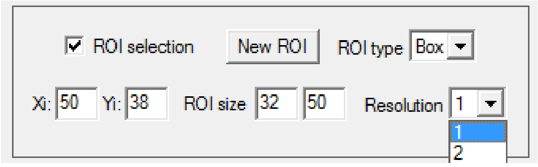


**Figure 10**. Detail of the main GUI showing the ROI selection options. When the <ROI selection> check box is checked, the user can select the ROI type, size as well as its resolution and top-left voxel coordinates.

If the check box `[ROI selection]` is checked, right and left mouse buttons are used to manually place ROIs in the display image and z slice. The drawing process depends of the type of ROI selected in the drop list `[ROI type]`. The ROI can be deleted in every moment using the button `[New ROI]`. The available ROI types include *Box*, *Full* or *free-type*.

- **Box ROI:** Square ROI. The upper left voxel of the ROI is selected y pressing the left window over the display image, or alternatively, typing the X and Y coordinates in the editable text fields `[Xi]` and `[Yi]`. The ROI size (in pixels) is decided using editable text field `[ROI size]`
- **Full ROI:** Using this option, the whole image slice is selected as ROI.
- **Free ROI**: This option allows selecting a free-form closed ROI. The user can draw the contour holding down the left mouse button, or defining line segments by pressing and releasing the left mouse button. Pressing the left button again, or pressing the `[New ROI]` button, erases the current region and the process starts.

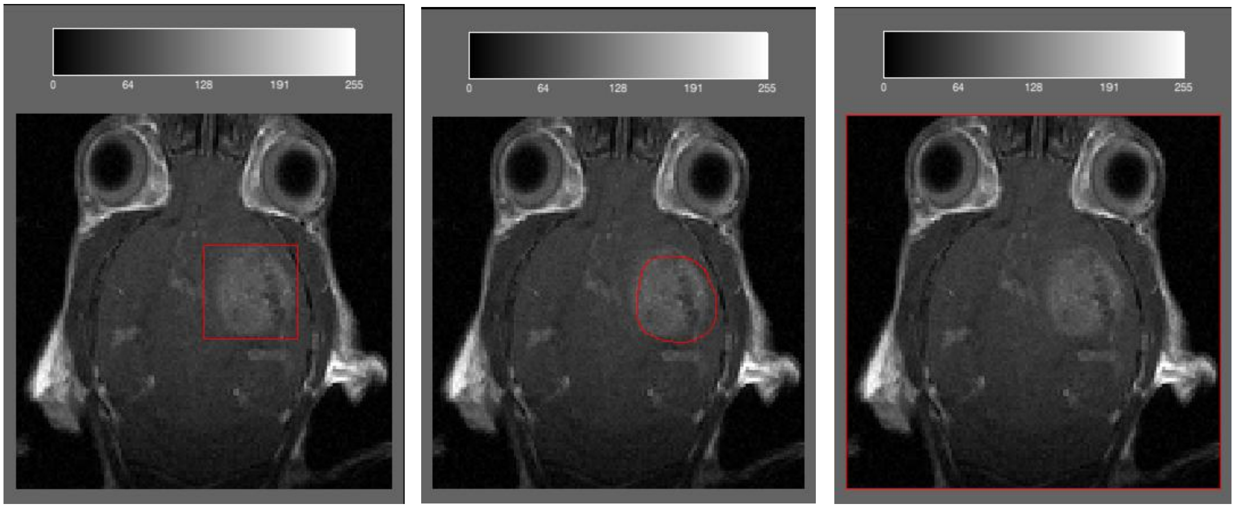


**Figure 11**. Example of ROI selection of box type, free type and full image type. The user can select three main shape types of ROIs: Irregular or "free" shape (shown at the left of the figure), box type (at the center of the figure) and whole image type, were voxels of the current slice are processed (at the right of the figure).

The user must also select the `[Resolution]` drop-down list, which selects the pixel size processed.

## Input parameters

To obtain correct results when the selected ROI is processed, processing parameters must be checked before each analysis. Input parameters are organized in tabs in the lower left corner of the main interface, as is shown in Figure 12.

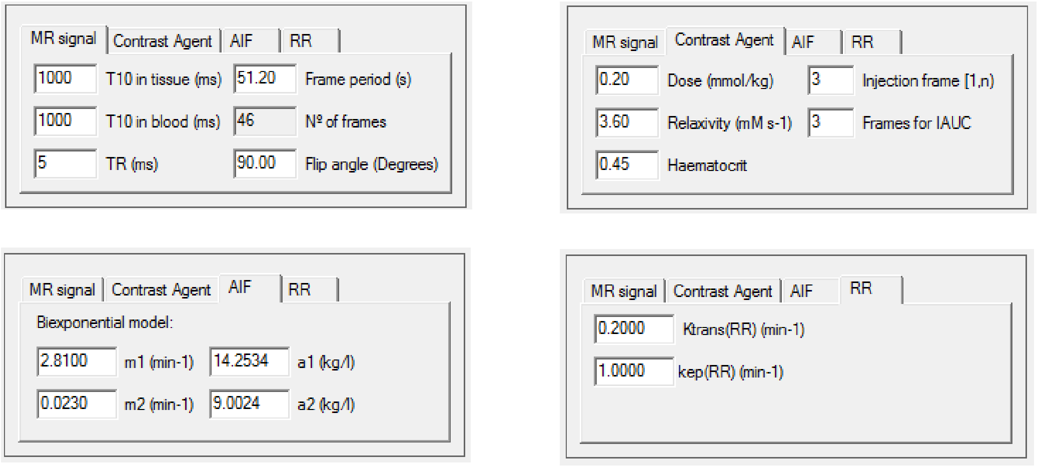


**Figure 12**. Tabs showing input parameters: MR signal tab, Contrast Agent tab, AIF (Arterial Input function) tab and RR (Reference Region) tab

Each tab groups a set of related parameters. The user must check the **MR signal** tab, **Contrast Agent** tab **AIF** (Arterial Input function tab) tab and **RR** (Reference region) tab. Depending of the pharmacokinetic model applied, the parameters will be effectively used or will not be necessary in the model. For further details about which parameters affects to each model please consult the "*DCE@urLAB_methods.pdf*" document.

### *MR signal tab*

Includes data related with the magnetic resonance (MR) acquisition protocol:

- `[T10 (ms)]` Longitudinal relaxation time prior to the injection of contrast agent, in milliseconds. Reference value for water.
- `[TR (ms)]` Repetition time of MR acquisition, in milliseconds. This value can be read automatically from the Bruker format header.
- `[Frame period (s)]` Time between temporal frame acquisitions, in seconds.
- `[Nº of frames]` Number of temporal frames. This data cannot be edited.

### *Contrast agent tab*

Under this tab are grouped the parameters associated with the contrast agent (usually Gd-DTPA)

- `[Dose (mmol/lk)]` Injected dose of contrast agent, in millimol/kilogram units.
- `[Relaxivity (mM s-1)]` T1 linear relaxivity of contrast agent, measured in milllimolar/seconds.
- `[Injection frame]` Dynamic frame when contrast agent was injected.
- `[Frames for IAUC]` Number of dynamic frames, counting from the injection frame, which is used in the calculus of the IAUC (Initial area under curve) parameter.

### *AIF agent tab*

This tab groups the parameters that describe the arterial input function (AIF) with a bi-exponential model (consult the "*DCE@urLAB_methods.pdf*" document for further details)

- [m1 (min-1)] Rate constant of exponential #1, in minutes$^{-1}$
- [m2 (min-1)] Rate constant of exponential #2, in minutes$^{-1}$
- [a1 (kg/l] Amplitude constant of exponential #1 in kilogram/liter.
- [a2 (kg/l] Amplitude constant of exponential #2 in kilogram/liter.

### *RR tab*

The RR tab includes the values of the reference region used in the RR model

- [Ktrans(RR) (min-1)] Volume transfer constant between plasma and EES, in minutes$^{-1}$
- [kep(RR) (min-1)] Rate constant between EES and plasma, in in minutes$^{-1}$

## Initial parameters

Dynamic curves are adjusted to pharmacokinetic models using iterative algorithms that require an initial set of parameters. These parameters can be changed opening the widget [Options-►Initial Parameters] in the bar menu. A new widget appears showing one tab by implemented model (Figure 10). Only in case of the optimization algorithm fails the user must try to change the initial values. RR model and modified Tofts model used the same initial parameters of Tofts model

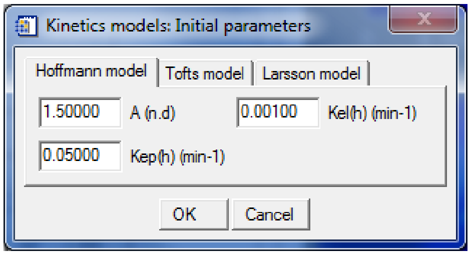


**Figure 13.** Initial values used in the optimization algorithms.

### *Additional information*

In the bar menu [View-►] the following options are available:

- [View dynamic images] Shows the whole dynamic frame with currently selected z slice. This option is not activated in the current version
- [View Cine] Shows a moving frame with the dynamic sequence of frames with the currently selected z slice.
- [View T1 map] Shows the current T1 map loaded in memory.
- [View ROI kinetic curve] Shows the mean value of ROI selected in the DCE-MRI dynamic signal.
- [View ROI kinetic curve (inc. SD)] Shows the ROI mean value and standard deviation (as error bars) of DCE-MRI dynamic signal.

# Export results

In the menu [Export/import] the user can select the following options[2]:

- [Export Images] The image displayed in the left or the right window is saved in Tiff format (24 bit color image). The image includes the palette bar.
- [Export ROI kinetic curve] A text file is saved with the RCE mean value of the whole ROI for every dynamic frame. The information is completed with the frame time and the standard deviation of the RCE within the ROI. The text is a three-column format separated with spaces.
- [Export ROI] The ROI morphology is saved in an IDL custom-format for later retrieval, allowing using the same ROI in different studies. A complimentary text file is saved in a two-column format, which contains the x and y coordinates of voxels included in the ROI. This file can be used to load the ROI in another software package. Finally, an image file (portable network graphics format) is saved with the mask of the ROI.
- [import ROI] The ROI file previously exported with compatible format is loaded in the platform.

The export of model analysis is performed from the results window and is described in the Export Results (Model analysis) section.

# Processing ROIs

If there is an active ROI selected, pharmacokinetic models and/or non-parametric analysis are estimated by pressing the button [Analyze ROI voxel-wise]. Note that this option is inactive until there is not a valid ROI.  The type of model analysis is selected with the drop-down list [Pharm. model]:

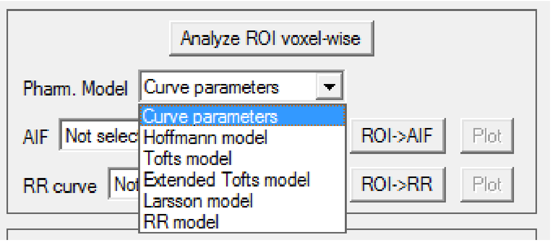


**Figure 14**. After selecting a valid ROI, the analysis button becomes active and the user can press the it to perform the model selected with the drop-down list

## *Pharmacokinetic modeling*

As it is shown in Figure 14 (left), **Hoffmann**, **Tofts**, **extended Tofts**, **Larsson** and **RR** model can be selected for analysis. Non parametric values (i.e., Curve parameters) are also included as an additional model.

[2] A dialog is opened to choose the name and location of the file.

Tofts and extended Tofts models need a valid AIF function, which is selected using the AIF drop-drown list. Valid options are:

- **Bi-exponential** The values of the AIF tab are used to calculate a bi-exponential AIF model. These values can be edited manually or by loading a proper configuration file in the bar menu: `[File–►Open AIF–►Parametric Function file]`
- **From file** The valid AIF should be loaded using the option `[File–►Open AIF–►Curve File]`
- **From ROI** the AIF selected using a ROI kinetic curve is used. This option is available using the button `[ROI–►AIF]` located next the drop-down list

The actual AIF used in the next pharmacokinetic model (if the model selected need that value) can be plotted using the button `[Plot]` located next the AIF options.

The RR model also need to have previously selected a valid RR curve, using the RR drop-down list. Avaliable options are :

- **From file** The valid RR curve should be loaded using the option `[File–►Open RR Curve File]`
- **From ROI** the RR is selected using a ROI. The button `[ROI–►AIF]` located next the drop-down list is used to this task.

The ROI can be analyzed again with different resolution, if value of `[Resolution]` drop list is changed[3]. An example of the result with box-type ROIs and 2 and 4 resolution parameter is shown in Figure 12.

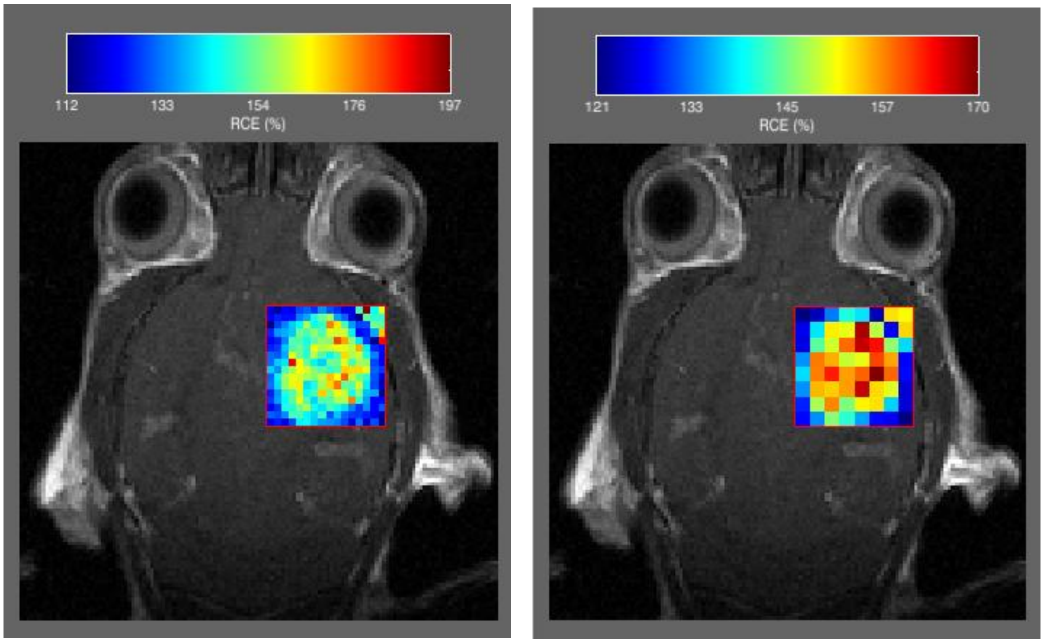


**Figure 15**. ROI analyzed with color map of selected parameter over the MRI data. The voxel resolutions were pharmacokinetic modeling is performed can vary in resolution: From intrinsic image resolution (the finest resolution) to coarse resolution. In the figure, two different coarse resolutions are shown

---

[3] Finest resolutions and large ROI require some processing time to adjust thousands of pixels.

## View and analyze results

After the analysis, which is performed pressing the button [Analyze ROI voxel-wise] and auxiliary window opens showing the voxel-wise values over the DCE-MRI data. Every time this option is selected, another results window appears so the user can analyze differences between models or parameters selected. An example of the results window is shown in Figure 16

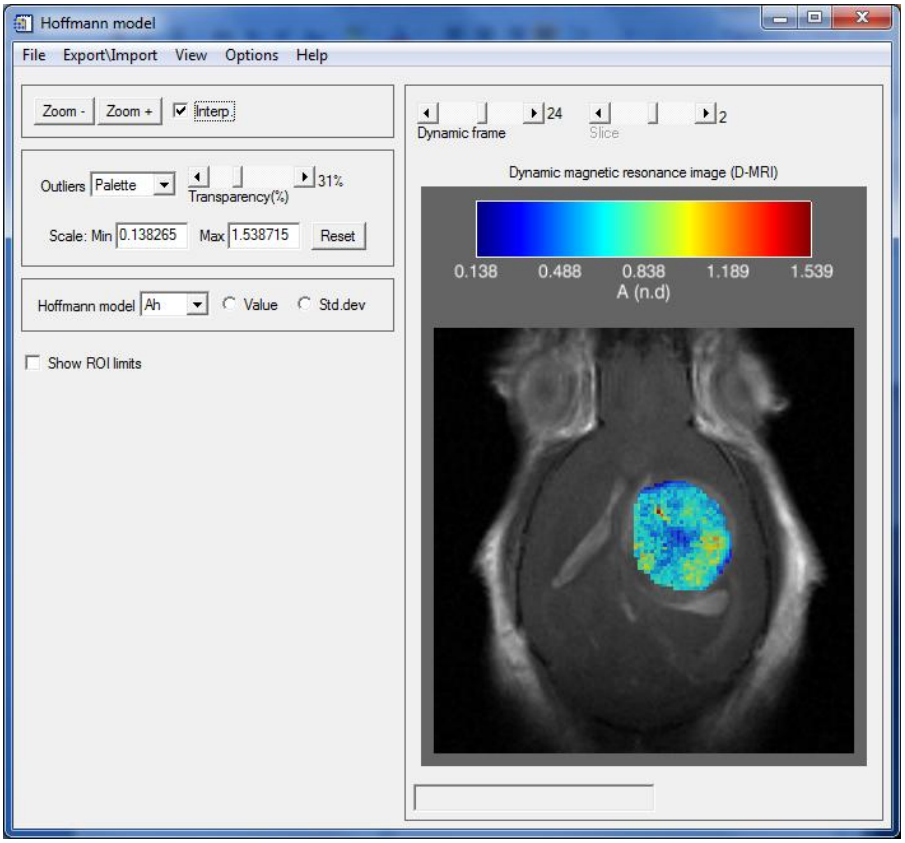


**Figure 16.** Results window with colormap of analyzed ROI superimposed over the DCE-MRI frame

### *Color maps*

Different parameters adjusted for each calculated model can be selected using the drop lists of Figure 11. The result is then overexposed on the MR image with a color palette that can be changed using the menu option [Options–►Color Palette–►ROI]. Additional visualization options are included in the left control menu:

- [Transparency] Sets the color transparency.
- [Scale: Min] and [Max] Set the maximum and minimum value of the palette. The scale for a certain parameter is remembered for the next time is selected.
  [Outliers] Drop list to decide how the values that are larger of smaller that the scale min and max are drawn.
- [Reset] Returns to the original scale between absolute values of the shown parameter.

When the mouse is situated over a ROI, the numerical value of selected parameter is shown in the information label (below the image).

### *Dynamic curves of single voxels*

If the left mouse button is pressed when the mouse is located over the ROI, the adjusted parametric model of the selected voxel is plotted together with the sampled MR signal[4]. An example of this plot is shown in Figure 17. The plot is completed with the estimated parameters of the model in the right side.

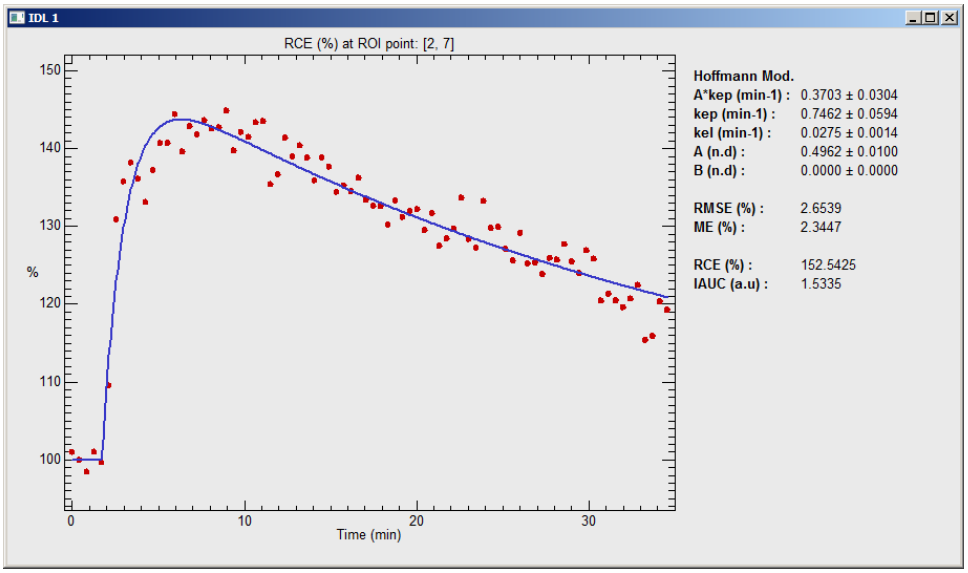


**Figure 17**. Dynamic signal and fitted model

### *Additional information*

In the menu [View-►] the following options are available :

- [View ROI model] Shows the selected pharmacokinetic modeled curve and its parameters corresponding to the ROI.

## Export results (pharmacokinetic modeling)

In the menu [Export/import] of the auxiliary results windows the user can select the following options[5]:

- [Export Image] The display image is saved in Tiff format (24 bit color image). The image includes the palette bar.
    - [Export ROI parameters] The active pharmacokinetic parameter map actually shown on the display image is saved in a text file. Three different formats can be selected:

---

[4] If more than one parametric model has been calculated, the model associated to the parameter selected is plotted.
[5] A dialog is opened to choose the name and location of the file.

- [Matrix format] The parameter of interest is saved in an ASCII text file with N files and N columns, where N is the ROI size divided by the voxel resolution in the X and Y dimensions; this format is only available with Box-type ROIs.
- [Single Column format] The actual parameter is stored in a single column, in a text file format. The order is compatible with the exported ROI format to be able to assign a voxel index to the values.
  [Multiple Column format] A multi-column file is saved with all parameters used in the selected model and its standard deviation, the root mean squared error (RMSE), the mean error in absolute value, and the voxel index in the X and Y dimensions

- [Export ROI kinetics] A text file is saved with the RCE mean value of the whole ROI for every dynamic frame. The information is completed with the frame time and the standard deviation of the RCE within the ROI. The text is a three-column format separated with spaces.
- [Export ROI] The ROI morphology is saved in an IDL custom-format for later retrieval, allowing using the same ROI in different studies. A complimentary text file is saved in a two-column format, which contains the x and y coordinates of voxels included in the ROI. This file can be used to load the ROI in another software package. Finally, an image file (portable network graphics format) is saved with the mask of the ROI.
- [import ROI] The ROI file previously exported with compatible format is loaded in the platform.

## Configuration file:

This sections describes the parameters included in the configuration file **DCEurLAB_config.txt** located in the DCE@urLAB main directory.

Each parameter contains a "**:=**" string separating the keyword and value. See Figure 15 for an example of configuration file. Comments must be preceded with symbol "##"

### General application parameters:

- **size_window** Size of the image windows, measured in pixels
- **Interpolation** [YES/NO]. Allow or deny linear interpolation in the image represented actually
- **Type_roi** [BOX/FREE/FULL]. Select the default option for the type of ROI
- **Size_roi** [x,y] Selects the size of the ROI of BOX type in x and y. The actual version only allows squared ROIS, so x must be equal to y
- **Resolution_roi** Selects the number of image pixels in the x direction that conform a ROI pixel for further analysis. Valid options are 1,2,3,4 and 8.
- **Path_data** Selects the default directory for reading DCE-MRI data and other input data
- **Parh_result** Selects the default directory where results and exported data are loaded when a dialog pickfile is opened.
- **Path_t1map** Selects the default directory for reading T1 maps.
- **Path_AIF** Selects the default directory for reading arterial input functions

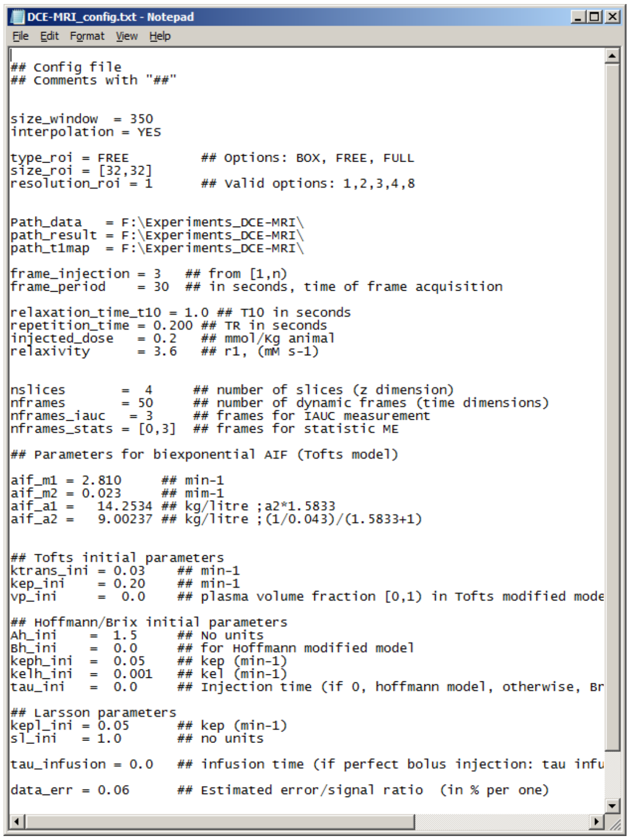

```
## Config file
## Comments with "##"


size_window  = 350
interpolation = YES

type_roi = FREE          ## Options: BOX, FREE, FULL
size_roi = [32,32]
resolution_roi = 1       ## Valid options: 1,2,3,4,8


Path_data   = F:\Experiments_DCE-MRI\
path_result = F:\Experiments_DCE-MRI\
path_t1map  = F:\Experiments_DCE-MRI\

frame_injection = 3   ## from [1,n)
frame_period    = 30  ## in seconds, time of frame acquisition

relaxation_time_t10 = 1.0 ## T10 in seconds
repetition_time = 0.200 ## TR in seconds
injected_dose   = 0.2   ## mmol/Kg animal
relaxivity      = 3.6   ## r1, (mM s-1)


nslices       =  4     ## number of slices (z dimension)
nframes       = 50     ## number of dynamic frames (time dimensions)
nframes_iauc   = 3     ## frames for IAUC measurement
nframes_stats = [0,3]  ## frames for statistic ME

## Parameters for biexponential AIF (Tofts model)

aif_m1 = 2.810     ## min-1
aif_m2 = 0.023     ## mim-1
aif_a1 =   14.2534 ## kg/litre ;a2*1.5833
aif_a2 =   9.00237 ## kg/litre ;(1/0.043)/(1.5833+1)


## Tofts initial parameters
ktrans_ini = 0.03    ## min-1
kep_ini    = 0.20    ## min-1
vp_ini     =  0.0    ## plasma volume fraction [0,1) in Tofts modified mode

## Hoffmann/Brix initial parameters
Ah_ini    =  1.5     ## No units
Bh_ini    =  0.0     ## for Hoffmann modified model
keph_ini  =  0.05    ## kep (min-1)
kelh_ini  =  0.001   ## kel (min-1)
tau_ini   =  0.0     ## Injection time (if 0, hoffmann model, otherwise, Br

## Larsson parameters
kepl_ini = 0.05      ## kep (min-1)
sl_ini   = 1.0       ## no units

tau_infusion = 0.0   ## infusion time (if perfect bolus injection: tau infu

data_err = 0.06      ## Estimated error/signal ratio  (in % per one)
```

**Figure 18.** Configuration file (DCE-MRI_config.txt)

## Data parameters:

- **Frame_injection** The number of temporal frame where the bolus injection of contrast is performed. If the study has N temporal frames, these are numbered from 1 to N
- **Repetition_time.** The repetition time (TR) of the T1 sequence, measured in seconds.
- **Injected_dose.** Relative dose of contrast agent injected in the study. The quantity is expressed in millimole/kg  mmol/kg)
- **Relaxivity** r1 relaxivity of the injected contrast agent. This parameter is measured in millimolar per second (mM/s) or mmol/ml/s.
- **haematocrit** HTC (haematocrit) value between 0 and 1.
- **t10_tissue** Precontrast tissue T1 (relaxation time) in seconds.
- **t10_blood** Precontrast blood T1 (relaxation time) in seconds.
- **flip_angle** Flip angle (in degrees) of spoiled gradient echo sequences (90 degrees in spin-echo sequences).

## Data parameters for read in raw mode

- **Frame_period.** Time of a single frame acquisition, or period between temporal frames, in seconds.
- **Nslices.** Number of pixels in the z dimension

- **Nframes.** Number of dynamic frames

## Measurement parameters:

- **Nframes_iauc**. Number of frames considered in the IAUC (initial area under curve) estimation.

## Parameters for biexponential arterial input function

- **Aif_m1, Aif_m2** Time constant of the exponentials of AIF function, expressed in min-1
- **Aif_a1, Aif_a2** Amplitudes of plasma curves in exponentials, expressed in kg/litre

## Initial parameters of Tofts model

Tofts model is optimized with a Levenberg-Marquardt algorithm that needs an initial estimation. Values too far away from final results could origin the non-convergence of the algorithm.

- **ktrans_ini** . Expressed in $min^{-1}$
- **kep_ini**. Expressed in $min^{-1}$
- **vp_ini** plasma volume fraction used in the modified Tofts model.

## Initial parameters of Hoffmann model

Brix model and it approximation Hoffman model are optimized with a Levenberg-Marquardt algorithm that needs an initial estimation of parameters. Values too far away from the true ones can produce a convergence to false values.

- **Ah_ini** Initial $A^h$ value in Hoffmann and Brix equations (see models reference). This parameter does not have units
- **Keph_ini** Initial $k_{ep}$ parameter (Efflux rate constant from EES to plasma) in $min^{-1}$
- **Kelh_ini** Initial $k_{el}$ parameter (clearance rate from plasma) in $min^{-1}$
- **Tau_ini** Initial time of infusion of contrast agent. This parameter is not used in this implementation and must be kept equal to zero.
- **Tau_infusion** Time of infusion of contrast agent, in $min^{-1}$. If a ideal bolus injection is administered (Tau_ini=0), then Hoffmann model is applied. Otherwise, Brix model is used. This parameter is not used in this implementation and must be kept equal to zero.

## Initial parameters of Larsson model

- **Kelp_ini** Initial $k_{ep}$ parameter (Efflux rate constant from EES to plasma) in $min^{-1}$
- **Sl_ini** Initial slope of the signal, without units.

## Parameters of reference region model

- **ktrans_rr** Value of ktrans of the reference region, in $min^{-1}$
- **ve_rr** Value of ve of the reference region, in $min^{-1}$

**Corresponding author:**

Juan Enrique Ortuño
Biomedical Image Technologies (BIT)
Dpto. de Ingeniería Electrónica, Universidad Politécnica de Madrid (UPM)
Av. Complutense "Ciudad Universitaria" nº 30, E-28040 Madrid (Spain)
e-mail: juanen@die.upm.es
web : www.die.upm.es/im

---

**DCE@urLAB**

DCE@urLAB
Copyright (c) 2012,2013, Universidad Politécnica de Madrid
All rights reserved.

Redistribution and use in source and binary forms, with or without modification, are permitted provided that the following conditions are met:

* Redistributions of source code must retain the above copyright notice, this list of conditions and the following disclaimer.
* Redistributions in binary form must reproduce the above copyright notice, this list of conditions and the following disclaimer in the documentation and/or other materials provided with the distribution.
* Neither the name of the Universidad Politécnica de Madrid nor the names of its contributors may be used to endorse or promote products derived from this software without specific prior written permission.

THIS SOFTWARE IS PROVIDED BY THE COPYRIGHT HOLDERS AND CONTRIBUTORS "AS IS" AND ANY EXPRESS OR IMPLIED WARRANTIES, INCLUDING, BUT NOT LIMITED TO, THE IMPLIED WARRANTIES OF MERCHANTABILITY AND FITNESS FOR A PARTICULAR PURPOSE ARE DISCLAIMED. IN NO EVENT SHALL UNIVERSIDAD POLITÉCNICA DE MADRID BE LIABLE FOR ANY DIRECT, INDIRECT, INCIDENTAL, SPECIAL, EXEMPLARY, OR CONSEQUENTIAL DAMAGES (INCLUDING, BUT NOT LIMITED TO, PROCUREMENT OF SUBSTITUTE GOODS OR SERVICES; LOSS OF USE, DATA, OR PROFITS; OR BUSINESS INTERRUPTION) HOWEVER CAUSED AND ON ANY THEORY OF LIABILITY, WHETHER IN CONTRACT, STRICT LIABILITY, OR TORT (INCLUDING NEGLIGENCE OR OTHERWISE) ARISING IN ANY WAY OUT OF THE USE OF THIS SOFTWARE, EVEN IF ADVISED OF THE POSSIBILITY OF SUCH DAMAGE.

**HCOLORBAR2.pro, FSC_Normalize.pro**

Copyright (c) 2008, by Fanning Software Consulting, Inc
All rights reserved.
Redistribution and use in source and binary forms, with or without
modification, are permitted provided that the following conditions are met:

* Redistributions of source code must retain the above copyright
notice, this list of conditions and the following disclaimer.
* Redistributions in binary form must reproduce the above copyright
notice, this list of conditions and the following disclaimer in the
documentation and/or other materials provided with the distribution.
* Neither the name of Fanning Software Consulting, Inc. nor the names of its
contributors may be used to endorse or promote products derived from this
software without specific prior written permission.

THIS SOFTWARE IS PROVIDED BY FANNING SOFTWARE CONSULTING, INC. ''AS IS'' AND ANY
EXPRESS OR IMPLIED WARRANTIES, INCLUDING, BUT NOT LIMITED TO, THE IMPLIED WARRANTIES
OF MERCHANTABILITY AND FITNESS FOR A PARTICULAR PURPOSE ARE DISCLAIMED. IN NO EVENT
SHALL FANNING SOFTWARE CONSULTING, INC. BE LIABLE FOR ANY DIRECT, INDIRECT,
INCIDENTAL, SPECIAL, EXEMPLARY, OR CONSEQUENTIAL DAMAGES (INCLUDING, BUT NOT LIMITED
TO, PROCUREMENT OF SUBSTITUTE GOODS OR SERVICES; LOSS OF USE, DATA, OR PROFITS
LOSS OF USE, DATA, OR PROFITS; OR BUSINESS INTERRUPTION) HOWEVER CAUSED AND
ON ANY THEORY OF LIABILITY, WHETHER IN CONTRACT, STRICT LIABILITY, OR TORT
(INCLUDING NEGLIGENCE OR OTHERWISE) ARISING IN ANY WAY OUT OF THE USE OF THIS
SOFTWARE, EVEN IF ADVISED OF THE POSSIBILITY OF SUCH DAMAGE.

---

**$Id: mpfitfun.pro,v 1.19 2012/09/27 23:59:31 cmarkwar Exp $**

Copyright (C) 1997-2002, 2003, 2006, 2007, 2009, 2010, 2011, 2012, Craig Markwardt
This software is provided as is without any warranty whatsoever.
Permission to use, copy, modify, and distribute modified or
unmodified copies is granted, provided this copyright and disclaimer
are included unchanged.

---

**$Id: mpfit.pro,v 1.82 2012/09/27 23:59:44 cmarkwar Exp $**

Original MINPACK by More' Garbow and Hillstrom, translated with permission
Modifications and enhancements are:

Copyright (C) 1997-2003, 2004, 2005, 2006, 2007, 2008, 2009, 2010, 2011, 2012 Craig Markwardt
This software is provided as is without any warranty whatsoever.
Permission to use, copy, modify, and distribute modified or
unmodified copies is granted, provided this copyright and disclaimer
are included unchanged.

---
