# Supplementary figures and images for: DCE@urLAB: a dynamic contrast-enhanced MRI pharmacokinetic analysis tool for preclinical data
Source: BMC Bioinformatics. 2013 Nov 4;14:316. doi: 10.1186/1471-2105-14-316 (PMC4228420; doi:10.1186/1471-2105-14-316)

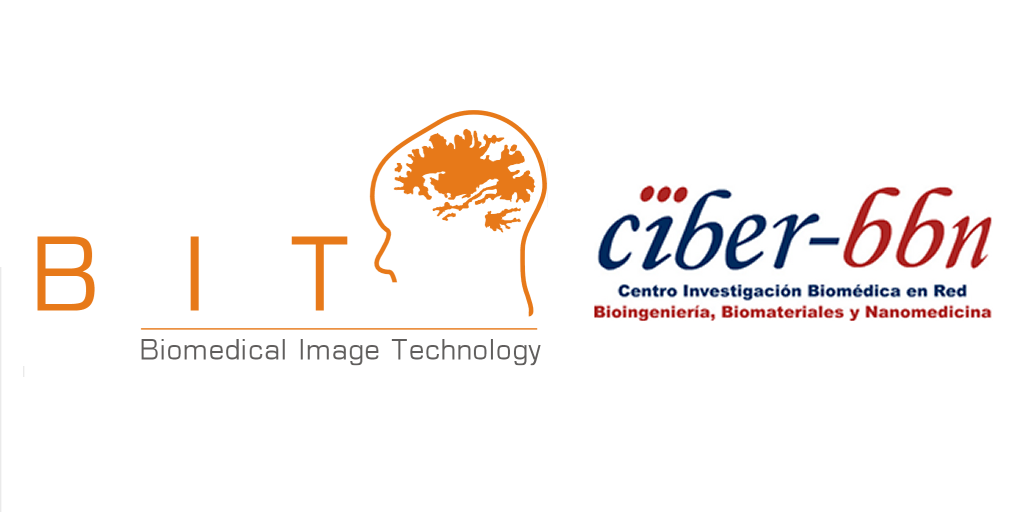

Supplement: Additional file 1 — Compressed file (zip format) with executable software, source code, and user manual. Unzip and read the file “/help/DCEurLAB_UserGuide.pdf” for instructions and details. [file 1471-2105-14-316-S1.zip › Icons/Logobit_ciber.png]

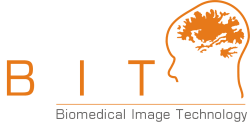

Supplement: Additional file 1 — Compressed file (zip format) with executable software, source code, and user manual. Unzip and read the file “/help/DCEurLAB_UserGuide.pdf” for instructions and details. [file 1471-2105-14-316-S1.zip › Icons/Logobit_small.png]

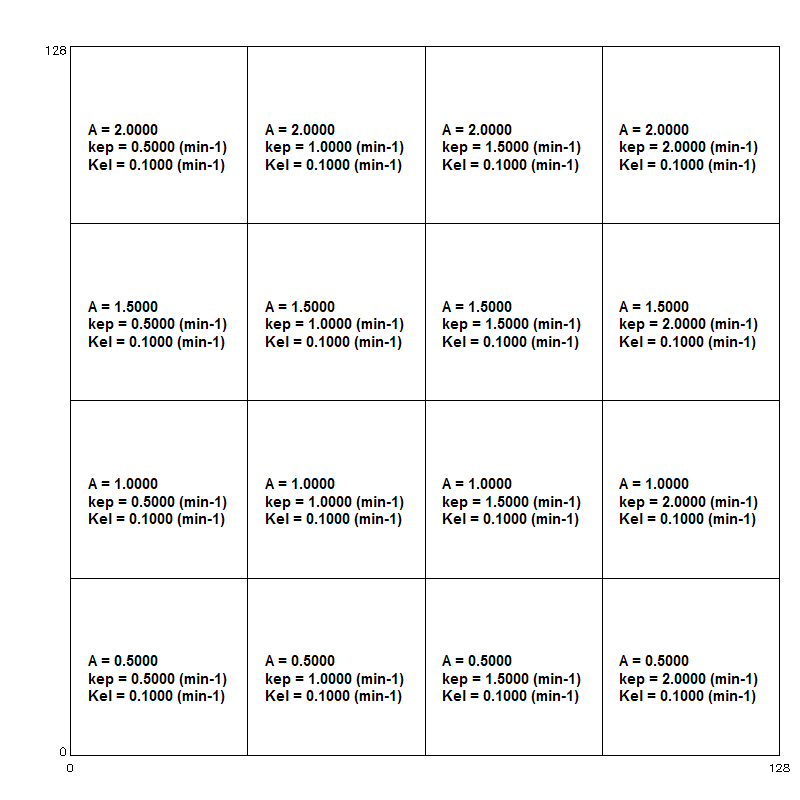

Supplement: Additional file 2 — Compressed file (zip format) with examples to test and validate the DCE@urLAB application. [file 1471-2105-14-316-S2.zip › Examples/Phantoms/Phantom_Hoffmann.png]

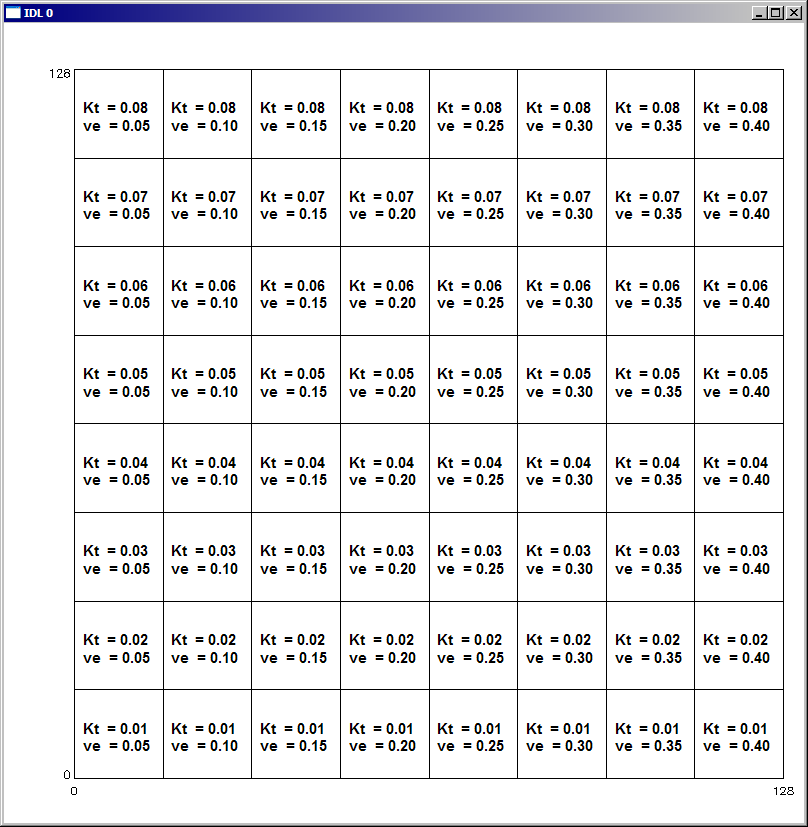

Supplement: Additional file 2 — Compressed file (zip format) with examples to test and validate the DCE@urLAB application. [file 1471-2105-14-316-S2.zip › Examples/Phantoms/Phantom_Tofts.png]
